# Supplementary material for: Impact of adherence to a lifestyle-integrated programme on physical function and behavioural complexity in young older adults at risk of functional decline: a multicentre RCT secondary analysis
Source: BMJ Open. 2022 Oct 5;12(10):e054229. doi: 10.1136/bmjopen-2021-054229 (PMC9535207; doi:10.1136/bmjopen-2021-054229)
Supplement: Author's manuscript [file bmjopen-2021-054229.draft_revisions.pdf]

BMJ Open is committed to open peer review. As part of this commitment we make the peer review history of every article we publish publicly available.

When an article is published we post the peer reviewers' comments and the authors' responses online. We also post the versions of the paper that were used during peer review. These are the versions that the peer review comments apply to.

The versions of the paper that follow are the versions that were submitted during the peer review process. They are not the versions of record or the final published versions. They should not be cited or distributed as the published version of this manuscript.

BMJ Open is an open access journal and the full, final, typeset and author-corrected version of record of the manuscript is available on our site with no access controls, subscription charges or pay-per-view fees (http://bmjopen.bmj.com).

If you have any questions on BMJ Open's open peer review process please email info.bmjopen@bmj.com

BMJ Open

Adherence to a Lifestyle-integrated Programme impacts physical function and behavioural complexity in young older adults at risk of functional decline – results from a multicentre RCT.

Journal:	*BMJ Open*
Manuscript ID	bmjopen-2021-054229
Article Type:	Original research
Date Submitted by the Author:	07-Jun-2021
Complete List of Authors:	Mikolaizak, A Stefanie; Robert-Bosch-Krankenhaus GmbH, Department of Clinical Gerontology<br>Taraldsen, Kristin; Norwegian University of Science and Technology (NTNU), Department of Neuromedicine and Movement Science<br>Boulton, Elisabeth; University of Manchester, School of Health Sciences; Manchester Academic Health Science Centre,<br>Gordt, Katharina; Robert-Bosch-Krankenhaus GmbH,<br>Maier, Andrea; The University of Melbourne, Department of Human Movement Sciences<br>Mellone, Sabato; University of Bologna, Department of Electrical, Electronic and Information Engineering «Guglielmo Marconi»<br>Hawley-Hague, Helen; University of Manchester, School of Health Sciences<br>Aminian, Kamiar; Ecole Polytechnique Federale de Lausanne<br>Chiari, Lorenzo; University of Bologna, Department of Electrical, Electronic and Information Engineering «Guglielmo Marconi»<br>Paraschiv-Ionescu, Anisoara; Ecole Polytechnique Federale de Lausanne<br>Pijnappels, Mirjam; Vrije Universiteit Amsterdam, Department of Human Movement Sciences<br>Todd, Chris; University of Manchester, School of Health Sciences<br>Vereijken, Beatrix; Norwegian University of Science and Technology (NTNU), Department of Neuromedicine and Movement Science<br>Helbostad, Jorunn<br>Becker, Clemens; RobertBoschKrankenhaus, Geriatric Medicine
Keywords:	Telemedicine < BIOTECHNOLOGY & BIOINFORMATICS, GERIATRIC MEDICINE, PUBLIC HEALTH, SPORTS MEDICINE

SCHOLARONE™
Manuscripts

I, the Submitting Author has the right to grant and does grant on behalf of all authors of the Work (as defined in the below author licence), an exclusive licence and/or a non-exclusive licence for contributions from authors who are: i) UK Crown employees; ii) where BMJ has agreed a CC-BY licence shall apply, and/or iii) in accordance with the terms applicable for US Federal Government officers or employees acting as part of their official duties; on a worldwide, perpetual, irrevocable, royalty-free basis to BMJ Publishing Group Ltd ("BMJ") its licensees and where the relevant Journal is co-owned by BMJ to the co-owners of the Journal, to publish the Work in this journal and any other BMJ products and to exploit all rights, as set out in our licence.

The Submitting Author accepts and understands that any supply made under these terms is made by BMJ to the Submitting Author unless you are acting as an employee on behalf of your employer or a postgraduate student of an affiliated institution which is paying any applicable article publishing charge ("APC") for Open Access articles. Where the Submitting Author wishes to make the Work available on an Open Access basis (and intends to pay the relevant APC), the terms of reuse of such Open Access shall be governed by a Creative Commons licence – details of these licences and which Creative Commons licence will apply to this Work are set out in our licence referred to above.

Other than as permitted in any relevant BMJ Author's Self Archiving Policies, I confirm this Work has not been accepted for publication elsewhere, is not being considered for publication elsewhere and does not duplicate material already published. I confirm all authors consent to publication of this Work and authorise the granting of this licence.

For peer review only

Adherence to a Lifestyle-integrated Programme impacts physical function and behavioural complexity in young older adults at risk of functional decline - results from a multicentre RCT.

Mikolaizak AS, PhD[1], Taraldsen K, PhD[2], Boulton E, PhD[3,4] , Gordt K, PhD[1], Maier AB, MD,PhD[5,6], Mellone S[7], PhD, Hawley-Hague H,PhD[3,4], Aminian K, PhD[8], Chiari L,PhD[7], Paraschiv-Ionescu A,PhD[8], Pijnappels M,PhD[5], Todd C, PhD[3,4,9], Vereijken B, PhD[2], Helbostad JL,PhD[2], Becker C,MD,PhD[1]

[1] Department of Clinical Gerontology, Robert Bosch Krankenhaus, Stuttgart, Germany; Stefanie.mikolaizak@rbk.de; katharina.gordt@issw.uni-heidelberg.de; Clemens.Becker@rbk.de

[2] Department of Neuromedicine and Movement Science, Norwegian University of Science and Technology (NTNU), Trondheim, Norway; kristin.taraldsen@ntnu.no; beatrix.vereijken@ntnu.no; jorunn.helbostad@ntnu.no

[3] School of Health Science, Faculty of Biology, Medicine and Health, University of Manchester, Manchester, UK; elisabeth.boulton@manchester.ac.uk; helen.hawley-hague@manchester.ac.uk; Chris.Todd@manchester.ac.uk

[4] Manchester Academic Health Science Centre, Manchester, UK

[5]Department of Human Movement Sciences, @AgeAmsterdam, Amsterdam Movement Sciences, Vrije Universiteit, Amsterdam, The Netherlands; m.pijnappels@vu.nl

[6]Department of Medicine and Aged Care, @AgeMelbourne, The Royal Melbourne Hospital, The University of Melbourne, Parkville, Vic., Australia; Andrea.Maier@mh.org.au

[7] Department of Electrical, Electronic and Information Engineering «Guglielmo Marconi», University of Bologna, Bologna, Italy; sabato.mellone@unibo.it;

For peer review only - http://bmjopen.bmj.com/site/about/guidelines.xhtml

lorenzo.chiari@unibo.it

[8] Laboratory of Movement Analysis and Measurement, Ecole Polytechnique Federale de Lausanne, Lausanne, Switzerland; kamiar.aminian@epfl.ch; anisoara.ionescu@epfl.ch

9 Manchester University NHS Foundation Trust, Manchester, UK.

Corresponding Author:

A. Stefanie Mikolaizak

Department of Clinical Gerontology, Robert Bosch Krankenhaus,

Stuttgart, Germany

Email: Stefanie.mikolaizak@rbk.de

Word count main text: 4025

Data elements: 4 tables

For peer review only

For peer review only - http://bmjopen.bmj.com/site/about/guidelines.xhtml

Abstract

Background

Long-term adherence to physical activity interventions is challenging. The Lifestyle-integrated Functional Exercise (LiFE) programme, was first adapted (aLiFE) to include more challenging activities and a behavioural change framework, and then enhanced (eLiFE) to be delivered using smartphones and smartwatches. We report an *a priori* subgroup analysis to 1) compare adherence measures, 2) identify determinants of adherence, and 3) assess the impact on outcome measures.

Methods: A multicentre, feasibility randomised controlled trial was conducted, comparing aLiFE and eLiFE against a control group. Participants aged 61-70 years from three European cities followed the interventions, reporting adherence monthly using a single question, and after six months intervention using the Exercise Adherence Rating Scale (EARS; Score range 6-24). Treatment outcomes included function and disability scores (measured using the Late-Life Function and disability Index) and sensor-derived physical behaviour complexity measure. Determinants of adherence were identified using linear multivariate analysis. Linear regression estimated the association of adherence levels on treatment outcome.

Results: 120 participants randomised to the intervention groups (aLiFE/eLiFE) (66.3±2.3 years, 53% females) are included. The 106 participants re-assessed after six months had mean EARS score of 16.0±5.1. Better adherence was associated with lower: number of medications taken, depression and risk of functional decline. We estimated adherence to significantly increase basic lower extremity function by 1.3 points ($p<.0001$), advanced lower extremity function by 1.0 points ($p<0001$) and behavioural complexity by .008 per one point higher EARS score ($F(3,91)=3.55$, $p=.017$); regardless of group allocation.

For peer review only - http://bmjopen.bmj.com/site/about/guidelines.xhtml

Conclusion: Physical activity adherence was associated with better lower extremity function and physical behavioural complexity. Barriers to adherence should be addressed pre-intervention, to enhance intervention efficacy. Further research is needed to unravel the impact of behaviour change techniques embedded into technology-delivered activity interventions.

Trial registration: NCT 03065088

Key words: physical activity, mobile health application, intervention, randomised controlled trial, behavioural change

Strengths and limitations of the study

- The intervention consisted of an activity programme integrated into daily life.
- Evidence-based behaviour change techniques were embedded into the intervention.
- Participants were from three European cities eliminating cultural bias towards the intervention.
- Adherence was defined *a-priori* to better capture intervention impact.
- Participants were randomised into treatment groups, rather than permitting patient preference.

For peer review only

Background

Exercise interventions conducted as randomised controlled trials are considered the gold standard for allowing causal interference to be made regarding the efficacy of a treatment. However, the reality is that the participants' adherence to treatment is typically imperfect. The actual intervention effect can only be evaluated accurately if adherence levels are high. Thus, conclusions on treatment effects of an exercise intervention will only be valid if the adherence levels to the protocol are evaluated [1, 2], with several studies reporting poor adherence rates among the study population to mediate the lack of treatment effect [3]. Further disentangling treatment (e.g. type of delivery mode) and exercise effect (e.g. type of exercise) on outcomes is essential when promoting benefits achieved through exercise interventions.

The World Health Organization defines adherence as the '*extent to which a person's behaviour corresponds with the recommendations from a healthcare provider*' [4] and it involves an active choice from the participant to follow through and to take responsibility for their well-being. In pharmaceutical trials, the intervention is 'unidimensional' and is based on patients taking the required dose, which can be monitored using bioanalytical methods. Interventions involving exercise and physical activity are more complex and 'multi-dimensional', as, for example, variance in daily form/fitness or required training effect influence the intensity required to maintain a specific training dose.

To date, there is no consensus regarding which adherence measurement tool should be used, nor when and how adherence should be measured [5, 6]. Besides the difficulty of recording adherence rates, achieving sufficiently high adherence is challenging. High adherence is frequently documented in the early stages of an exercise intervention, regardless of delivery mode and exact definition used [2, 3]. In contrast, long-term intervention adherence can be as low as 30% [2], with activity levels reverting to pre-intervention levels following the

For peer review only - http://bmjopen.bmj.com/site/about/guidelines.xhtml

intervention period. Integrating tailored activities into daily life help achieve longer-term adherence levels than a structured programme, where exercise is segregated from daily routines [7]. While this delivery method is gaining in popularity[8], there is limited knowledge on how to assess adherence to a lifestyle-integrated activity programme. Furthermore, a recent systematic review showed that interventions that used telecommunication, and those offering lifestyle-integrated exercise interventions, appeared most promising for maintaining adherence[3]. Despite this, interventions that specifically targeted adherence did not affect balance or gait parameters.

Adherence to exercise appears to be influenced by socioeconomic status, health, physical function, motivation and other psychological variables [9, 10]. Understanding which factors influence young older adults (60-70 years old) to adhere to lifestyle-integrated exercises is essential for future intervention success [3]. Modifiable factors that influence adherence need to be identified, ideally pre-treatment, in order to influence treatment effect.

This paper reports *a priori* secondary analysis of the PreventIT randomised controlled feasibility trial data to investigate factors influencing adherence to a personalised lifestyle-integrated exercise intervention offered to young older adults, with the intention to prevent accelerated functional decline. Here, adherence is defined as participants completing all their planned lifestyle-integrated activities into daily life, as described below. Further, adherence was hypothesised to be a treatment-effect modifier which needs to be analysed within the intervention groups. The current *a-priori* analysis based on adherence levels is warranted. It may have greater clinical relevance, be more indicative of the provided treatment effect [11], aid future improvements of the intervention programme, and guide implementation into practice.

For peer review only - http://bmjopen.bmj.com/site/about/guidelines.xhtml

Aims

The aims of this analysis are to: 1) assess whether measuring adherence monthly prospectively, and retrospectively every six months, yields the same results; 2) identify baseline characteristics that determine adherence levels, and 3) estimate the association of adherence to a lifestyle-integrated intervention on primary and secondary outcome measures (functional ability and behavioural complexity) in young older adults.

METHODS

Design

This *a-priori* subgroup analysis is part of a large-scale multicentre RCT - PreventIT (Clinical trial NCT 03065088), analysing the impact of a 6-month active intervention period within a 12-month trial. PreventIT, a personalised behavioural change exercise intervention for young older adults, was developed to prevent accelerated functional decline. The detailed protocol [12] and results of the feasibility analysis, including an estimate of change for the primary outcome measures have been published elsewhere[13]. Ethical approval was obtained the relevant ethical committees of the clinical sites (Norway (REK midt, 2016/1891), Stuttgart (registration number 770/2016BO1), and Amsterdam [METc VUmc registration number 2016.539 (NL59977.029.16)]. The patients/participants provided their written informed consent to participate in this study.

Participants

The trial sample for this pre-planned analysis includes the intervention group participants from the two treatment modes (n=120). Participants were invited to participate if they were aged 61-70 years, retired or working part-time, community dwelling and able to walk 500 meters without a walking aid. Exclusion criteria included participation in an exercise class (>1/week) or undertaking moderate-intensity physical activity (≥150 min/week).

Intervention

For peer review only - http://bmjopen.bmj.com/site/about/guidelines.xhtml

Participants received tailored exercises at an individual level and learned to integrate these into everyday situations. The same activity framework was applied in both intervention arms, and was delivered in two different modes: via paper-pen manuals (aLiFE) or via a mobile health application system (eLiFE). Participants received several home visits from an exercise physiologist/scientist or physiotherapist during which the exercise program was taught. The programme consisted of strategies to a) improve balance; b) increase muscle strength; and c) reduce sedentariness and increase physical activity (PA). The concept, based on the LiFE programme [7], has been adapted to young older adults to be more challenging [14], and is underpinned by a behavioural change framework to support older adults to form long-term physical activity habits. PreventIT has taken the original LiFE concept and further developed the behaviour change elements, explicitly mapping them to Social Cognitive Theory, Habit Formation Theory, and 30 Behaviour Change Techniques (BCTs)[15] Goal setting, planning, prompts, and real-time feedback are used to deliver a person-centred experience.

Participants received their randomly allocated intervention during the first six months (active period) of the programme and were then encouraged to keep up their programme (passive period) independently for another six months. Here, an analysis of the first 6-month active intervention period is presented.

For context, control group participants received general WHO advice on the benefits of physical activity but no tailored exercise program. Therefore an exercise adherence analysis was not relevant for this group.

Exercise adherence was encouraged in general to all participants and participants were provided with adherence logs to document their activity. No incentives or additional adherence promotion was provided to the participants within the PreventIT trial.

Outcome measures

For peer review only - http://bmjopen.bmj.com/site/about/guidelines.xhtml

Participants underwent a comprehensive assessment by an assessor blinded to group allocation at baseline, after a 6-month active, and a further 6-month passive intervention period.

Adherence measures

Adherence was collected using two methods in this trial.

Exercise Adherence Reporting Scale at post-test and follow-up

The Exercise Adherence Reporting Scale (EARS) [16] was completed by participants during follow-up as an objective, reliable, and validated self-report measure of adherence to planned (exercise) activities. EARS consists of 16 questions, each answered on a 5-point Likert-scale (scored 0-4), identifying how strongly the participant agrees/disagrees with each statement. Six questions capture the level of adherence, while 10 questions address facilitators or barriers of adherence. A higher score indicates better adherence; however, a pre-defined cut-off point for adequate adherence does not exist. Adherence is frequently dichotomised, based on an arbitrary cut-off, however, adherence with an ongoing complex treatment is variable, and analysing adherence as a continuous or ordinal variable is warranted [17].

Monthly reports of adherence

Every month, participants were asked to report whether they had completed their planned activities 'more than planned', 'as much as planned', 'less than planned' or 'not at all' because i) 'they did not feel well', ii) 'they forgot', iii) 'they did not like the activities' or iv) 'they did not have time'. Response to the single question was possible via email or postcard where each month of reporting was summarized as full adherence (responded positively, and "all or more than planned"), partial adherence (responded positively, and "but not as much as planned"), or non-adherence (remaining responses). Data from participants who provided four or more responses during the six-month intervention and follow-up period were included in the analysis.

For peer review only - http://bmjopen.bmj.com/site/about/guidelines.xhtml

The validity of this novel method of assessing adherence was tested within the PreventIT feasibility trial [13].

Treatment outcome measures

The trial included two primary outcome measures [12]: subjective health rating using the Late-Life Function and Disability Index (LLFDI) [18], and physical activity measure using a behavioural complexity metric [19].

The LLFDI is a comprehensive questionnaire assessing function (ability to perform specific activities of daily living) and disability (inability to take part in major life tasks and social roles) for use in community-dwelling older adults [18, 20].

The behavioural complexity metric was derived from a 7-day consecutive physical activity monitoring period, and assessed in the domains of daily physical activity, and social participation. A 3-Axis Logging Accelerometer (https://axivity.com/product/ax3) attached to participants' lower back (using adhesive tape) represent physical behaviours as time series embedding activity characteristics (i.e., type, duration, intensity, and dynamics of transitions between activities) [19, 21] . The concept of behavioural complexity, initially developed to assess physical behaviour in chronic pain patients [21, 22], was further developed in the PreventIT trial using novel computational complexity methods, and the relationship of complexity metrics with additional clinical scores/outcomes, such as fear of falling, and functional balance and mobility performance pre- and post-intervention [19, 23].

Additional outcome measures included general health and function, medication use, neuropsychological assessment, physical function and risk of functional decline (Table 1). The PreventIT risk screening tool was used to identify and provide a participant's risk estimate (low, medium, or high risk) for the functional decline over the next nine years [24].

For peer review only - http://bmjopen.bmj.com/site/about/guidelines.xhtml

Determinants of adherence

Key factors which were anticipated to positively or negatively influence participants' adherence were selected from the outcome measures obtained during the PreventIT baseline assessment [13]. Determinates were chosen if they were (i) known functional decline risk factors (e.g. age, or number of medications); (ii) strength or balance deficits, identified during assessment; (iii) potentially associated with adherence (e.g., pain when walking, cognitive impairment) (Table 3) [12].

Statistical analysis

Descriptive statistics were used to summarise demographic data. Baseline comparisons depending on adherence levels were performed using parametric and nonparametric tests as appropriate. Initially, the association between adherence, as reported monthly during the active intervention period (0-6 months post randomisation), and EARS sum score of Question 1 to Question 6, reported at six months post-randomisation, was assessed using Pearson's correlation. In further analyses the continuous EARS score was used.

The modal response was used as an imputation method to account for missing monthly reporting data, which was missing completely at random, allowing 106 participants (53 from each treatment arm) to be included in univariate/multivariate analysis to reduce bias [25].

Univariate associations between potential determinants and adherence (EARS score) were assessed using logistic regression. Variables identified during univariate regression ($p<0.05$) were subsequently entered into a multivariate linear regression model.

Multiple linear regression was used to estimate the association of predicted adherence on primary and secondary outcome measures at six months follow-up, using group allocation (aLiFE and eLiFE), baseline values, and predicted adherence levels as independent variables. Participants' predicted adherence levels (EARS score) were derived using multiple linear

For peer review only - http://bmjopen.bmj.com/site/about/guidelines.xhtml

For peer review only

regression results [26, 27]. Predicted adherence levels were centred [28] and the interaction association between group allocation and adherence level is presented only when significant. Main associations of baseline values, group allocation and adherence levels are presented. Analyses were conducted using SPSS version 25.0 (SPSS Corp).

RESULTS

Fifty-nine participants were randomised to receive the traditional paper-pen based (aLiFE) and 61 the technology supported (eLiFE) programme. The six months follow-up was completed by 53 (89.8%) aLIFE and 53 (86.9%) eLIFE participants. Participant characteristics are presented in Table 1. Mean age of participants was 66 years (SD: 2.3) and an equal number of male and female were enrolled. Most participants had not experienced a fall in the past year, were taking two medications daily and were overall in good health.

[INSERT TABLE 1 HERE]

Monthly and six-monthly adherence measurement

During the six months follow-up period, which also included monthly follow-up, 26 (24.5%) participants reported fully adhering to their planned activity, whilst 21 (19.8%) participants reported non-adherence (Table 2). The remainder (59, 55.7%) were classified as partial adherers. During the six-months follow-up assessment, the mean EARS score was 16.02 (SD: 5.12). These results showed a large, positive, and significant association between the monthly adherence reporting and the EARS questionnaire score at six months ($r(102)= .618$, $p<0.001$).

[INSERT TABLE 2 HERE]

Determinants of adherence

For peer review only - http://bmjopen.bmj.com/site/about/guidelines.xhtml

Univariate analyses identified nine baseline variables to be associated with intervention adherence using EARS (Table 3), of which three variables were retained in the multivariate linear regression model: Greater number of medication (Beta = -.21, p = 003), CES-D score (Beta = -.25, p = .009), and the risk of functional decline score (Beta = -.21, p = .35) all significantly influenced poorer adherence levels.

[INSERT TABLE 3 HERE]

<u>Estimated association of adherence on outcome measures</u>

After controlling for group allocation, predicted adherence level was positively associated with improved physical function, behavioural complexity and well-being (Table 4) at follow-up. Group allocation was not significantly associated with an improvement in any primary or secondary outcome measure.

Treatment outcomes

Higher predicted adherence levels were significantly associated with improved LLFDI scores. In summary, one EARS point higher resulted in 0.67 point improvement on self-reported disability limitation (higher score indicates less perceived limitation) (F(3,102)=42.50, p<.001). An improvement in all LLFDI function subcomponents was significantly associated with improved adherence. For each EARS point higher, upper extremity (UE), basic lower extremity (BLE), and advanced lower extremity (ALE) function improved by 1.11 points (F(3,102)=35.90, p<.001), 1.30 points (F(3,102)=46.60, p<.001), and 1.0 points (F(3,102)=90.07, p<.001), respectively.

For peer review only - http://bmjopen.bmj.com/site/about/guidelines.xhtml

Behavioural complexity increased by 0.08 per point increase in EARS score; however, this change was not significant.

CES-D scores was reduced by 0.89 points ($F(3,102)=28.53$, $p<.001$), while walking speed increased by 0.014m/s ($F(3,102)=50.28$, $p<.001$) per point increase in EARS score.

[INSERT TABLE 4 HERE]

Discussion

In this analysis we have demonstrated that within this cohort, monthly reporting of adherence (prospective) correlated strongly with a single retrospective report of adherence (EARS) after a six-month intervention period. We identified three main factors that influenced young older adults' adherence to their planned activity: the number of medications they were taking, their level of depression, and their risk score of accelerated functional decline. Higher predicted adherence levels had a significant and clinically meaningful benefit on self-reported function (LLFDI) and greater behavioural complexity. Further, predicted adherence was significantly associated with improved gait speed and lower depression scores. To our knowledge this is the first analysis that examines the association of adherence to a lifestyle-integrated exercise programme with the intention to prevent functional decline in young older adults.

Measuring adherence

To date, there is no consensus on how adherence should be measured. Of the existing approaches [6] a non-standardised, arbitrary binary cut-off is commonly used to categorise participants into 'adherers' and 'non-adherers'. This approach has two main flaws. First, it fails to acknowledge that adherence is a complex construct of continuous quantity [17]. Second, dichotomising participants based on an arbitrary cut-off assumes that partial adherence has a

For peer review only - http://bmjopen.bmj.com/site/about/guidelines.xhtml

nil treatment effect, when in fact some adherence may affect the dependent outcome measure [29]. Even not completing activities as often or as intended, any activity is still breaking up sedentary behaviour. Within this trial, we used monthly reporting of adherence, building on the gold-standard for prospective fall data collection [30], to eliminate recall bias due to delayed reporting. Further, we used the validated EARS questionnaire after the 6-month active intervention period. We found strong and highly significant correlation between the monthly (prospective) and the bi-annual (retrospective) responses, indicating that a single measurement time-point could suffice during the active intervention period. Ultimately, the method chosen to measure adherence should be determined by its purpose [6].

Monthly adherence reporting allows for tracking the change in adherence over time. From an intervention perspective, this could be used as a facilitator to identify adherence problems early on and address potential barriers in a timely fashion. However, monthly adherence reports are also a form of intervention in themselves, as they acted as reminders to complete the planned activities [13]. Regular prompting has been shown to improve adherence [31], and prompts in themselves are part of the BCT taxonomy [32]. Further, frequent reporting could also be considered burdensome on the participant, and a single retrospective questionnaire may be preferred. Within our young older population using EARS every six months seems sufficient to measure adherence accurately when the purpose of adherence documentation is to obtain an average score per participant, following a six-month intervention period.

Determinants of adherence

Adherence to the lifestyle-integrated activity programme varied among participants, with participants reporting on average 16 out of 24 points on EARS, indicating agreeable and positive intent to adhere. Such variability in adherence has repeatedly been reported for exercise interventions involving older adults [2] and is slightly higher than reported elsewhere [16].

For peer review only - http://bmjopen.bmj.com/site/about/guidelines.xhtml

For peer review only

Previously, it has been demonstrated that external factors impact adherence behaviour such as location, frequency of exercising, level of supervision and delivery mode [33]. Also, there are numerous patient-specific, internal factors that directly or indirectly appear important for adherence behaviour [9, 10]. Within our study population, the number of medications used, depression level and risk of accelerated functional decline were negatively associated with adherence. Increased medication use could be seen as a proxy for comorbidities, a barrier for adherence [34]. Surprisingly in our sample, the presence of arthritis or pain while walking was not an adherence barrier, as described in other studies [35]. However, reported pain levels were low, and bias from under-enrolment of those with severe arthritis is possible.

While exercise is an effective intervention to manage depression [36], depression adversely impacts adherence to exercise interventions, as shown here and in other studies [37]. Measuring baseline depression levels as an established adherence barrier predict adherence before intervention studies and thus tailoring the intervention and support accordingly.

The third factor which impacted adherence in this study was risk of accelerated functional decline. Those at high risk were limited by the burden of already deteriorating health, as shown previously [10], while those at moderate risk may have understood the urgency to undertake preventative measures and were still capable of doing so [38]. Positive effects of education and awareness of adherence have shown in exercise programmes in participants with cardiovascular conditions [39]. Participants within our trial were informed of their risk profile and had undergone detailed assessment, which highlighted their balance and strength deficits, and could have acted as a 'prompt' to adhere to the intervention.

When enrolling participants in an intervention programme, assessing physical and psychological adherence barriers can help tailor the intervention delivery to suit participants'

For peer review only - http://bmjopen.bmj.com/site/about/guidelines.xhtml

preferences and needs [37]. This provides an opportunity to identify non-adherers early and target known barriers, for example, by applying behaviour change techniques to address psychological barriers, such as poor self-efficacy or poor motivation (or depression) [31], or by multidisciplinary interventions to manage pain and comorbidities better.

Estimated association of adherence

Adherence to an intervention programme influences the intervention dose, which for physical activity interventions can lead to an improvement in physical outcome measures [17, 40]. Predictive modelling showed that higher adherence levels were significantly associated with improved subjective health and function (LLFDI), as well as with improved behavioural complexity scores, gait speed and depressive symptoms. These results are in line with the previous reporting of balance training improving self-reported function in community-dwelling older adults [41], as well as exercise improving depression levels [42] and increasing gait speed [43]. However, several other physical outcome measures remained unchanged, despite higher estimated adherence levels. These inconclusive findings are similar to those by Hughes et al. [3], who demonstrated that even interventions that focus specifically on improved adherence did not achieve meaningful change in clinical outcome measures. Future trials need to understand who within their patient population will adhere, and why, and which adherence barriers can be addressed and modified.

The analysis presented here spans the first six months of the intervention period. Overall, the adherence rates were low, with an average EARS score of 16.75 points at six months in the eLiFE group and 15.29 in the aLiFE group. These results are in line with findings from other technology-supported trials, which have shown adherence rates to drop as time passes, with only 25% of participants still adhering at the end of a 48-week home-exercise programme [44].

For peer review only - http://bmjopen.bmj.com/site/about/guidelines.xhtml

The slightly higher adherence rates in eLiFE could be due to the functionalities available within the app.

Within the intervention period, participants in eLiFE and aLiFE received four or six home visits respectively. Participants were encouraged to integrate their activities into everyday routines, without supervision. Although unsupervised activities have been shown to improve self-reported adherence [45], this did not translate into an improvement in clinical outcome measures. A possible explanation is that while better adherence is reported, the quality, accuracy, and intensity of the activity is not ideal when unsupervised, limiting its benefits. Further work incorporating technological measures of adherence are needed to document accurately the quality and fidelity of the activity undertaken in an unsupervised setting. Beyond documenting exercise execution, an ICT platform could provide feedback to participants about how and what to improve during the intervention programme. Simultaneously, health care providers and researchers could access this information and use telecommunication to increase adherence, optimise exercise accuracy/quality, and increase the likelihood of achieving an improvement in clinical outcomes.

Strength and limitations

A strength of this study was that the activity programme offered to the participants was not detached from daily life, but instead integrated into existing routines. Providing an opportunity to be more active has been labelled a key element to achieving better adherence rates both with [46] and without [7] the use of technology. Within this trial, the intervention was personalised and adapted to the individual's capabilities, similar to approaches by other researchers who individualised interventions integrated into existing routines [47, 48]. Within our study [15], different behaviour change techniques were applied and combined to improve adherence , as this has been identified as an essential intervention component to promote sustainable changes

For peer review only - http://bmjopen.bmj.com/site/about/guidelines.xhtml

in activity habits [32]. Future studies should evaluate technology-delivered methods which apply different behaviour change techniques to unravel the added benefit of both components. During analysis, adherence was treated as a continuous variable, which fulfils the fifth assumption (exclusion restriction) in so called "complier average causal effect analysis" [26, 27, 49]. Using the continuous EARS score to quantify adherence can capture the effect of proportionate adherence.

Several limitations of this study are acknowledged. The estimate of the association of adherence only allows us to determine participation levels are associated with better outcomes and it does not estimate the causal effects of the treatment since there is no comparison with a comparable group. As the intention-to-treat analysis showed no difference in effect between groups, a suitable adherer "average causal effect analysis" was not possible. Two different treatment modes are compared based on adherence levels. It was not possible to assess whether adherence to eLiFE has greater implications on clinical outcomes than adherence to aLiFE. However, both intervention modes apply the same activity framework, and analysis to determine the estimated effect of adherence was defined *a priori*. Future work should consider further subgroup analysis to understand the adherence to different intervention methods better.

Implications for future research and clinical practice

Group allocation did not impact adherence in this study; however, research [50] has shown that adherence can be increased when patients can select the intervention mode. Offering patients a choice of intervention (patient preference) it could impact adherence rates without compromising evidence-based interventions [51].

For peer review only - http://bmjopen.bmj.com/site/about/guidelines.xhtml

To encourage higher rates of adherence, barriers such as depressive symptoms or polypharmacy, could be addressed as part of a pre-intervention to enhance the ability to test new interventions.

In future trials, we should consider measuring baseline characteristics that affect adherence levels. This may include variables not generally considered for standard data collection, such as social support to support interventions, what exercise programmes have previously been explored, and outcome expectations [15]. With improved baseline prediction of adherence and patient preference interventions, subsequent interventions can be more accurately assessed.

Funding: This work was supported by the EU funded project PreventIT (2016–2018, grant number 689238) responding to the Horizon 2020, Personalized Health and Care call PHC-21: Advancing active and healthy aging with ICT: Early risk detection and intervention. The EU was not actively responsible or involved in the study design, collection, management, analysis, or interpretation of data. The writing of reports and the decision to submit for publication is not authorized by the EU.

Acknowledgement: We thank all the participants in the trial, additional medical staff, assessors and instructors at the three clinical sites, and all partners in the consortium for their support, assistance, and input throughout the different phases of the project.

Author Contribution: KT, EB, HHH, ABM, SM, KA, LC, MP, CT, BV, JH and CB designed research; ASM, KT, EB, HHH, KG and AP conducted research; ASM, KR, KG and AP analysed data, performed statistical analysis and interpreted outcomes; ASM, KT and EB

For peer review only - http://bmjopen.bmj.com/site/about/guidelines.xhtml

prepared the manuscript; ASM, KT, EB, BV, JH and CB had primary responsibility for final content; all authors read and approved the final version of the manuscript.

Data statement

The datasets presented in this article are not readily available because the datasets for this study are not open accessible to all, but only shared between project partners due to ethical approval guidelines in the three clinical sites conducting the multicenter trial, and cannot be shared until 10 years after the end of the project. Requests to access the datasets should be directed to kristin.taraldsen@ntnu.no.

Conflicts of interests

SM and LC own a share in the spin-off company of the University of Bologna, mHealth Technologies srl. AZ was funded by the Doxee s.p.a. during the conduction of the trial, and by CINECA after the end of the project.

The remaining authors declare that the research was conducted in the absence of any commercial or financial relationships that could be construed as a potential conflict of interest.

Patient and Public involvement

Participants from within the target group were involved in the design of the eLiFE app during the pilot study which formed part of PreventIT.

For peer review only - http://bmjopen.bmj.com/site/about/guidelines.xhtml

References

1. Sherrington, C., et al., *Exercise to prevent falls in older adults: an updated systematic review and meta-analysis.* British journal of sports medicine, 2016. **ePub**(ePub): p. ePub-ePub.
2. Nyman, S.R. and C.R. Victor, *Older people's participation in and engagement with falls prevention interventions in community settings: an augment to the Cochrane systematic review.* Age Ageing, 2012. **41**(1): p. 16-23.
3. Hughes, K.J., et al., *Interventions to improve adherence to exercise therapy for falls prevention in community-dwelling older adults: systematic review and meta-analysis.* Age Ageing, 2019. **48**(2): p. 185-195.
4. Organisation, W.H., *Adherence to long-term therapies*. 2003: Geneva.
5. Aronson, J.K., *Compliance, concordance, adherence.* Br J Clin Pharmacol, 2007. **63**(4): p. 383-4.
6. Hawley-Hague, H., et al., *Review of how we should define (and measure) adherence in studies examining older adults' participation in exercise classes.* BMJ Open, 2016. **6**(6): p. e011560.
7. Clemson, L., et al., *Integration of balance and strength training into daily life activity to reduce rate of falls in older people (the LiFE study): randomised parallel trial.* BMJ, 2012. **345**: p. e4547.
8. Weber, M., et al., *Feasibility and Effectiveness of Intervention Programmes Integrating Functional Exercise into Daily Life of Older Adults: A Systematic Review.* Gerontology, 2018. **64**(2): p. 172-187.
9. Mikolaizak, A.S., et al., *Adherence to a multifactorial fall prevention program following paramedic care: Predictors and impact on falls and health service use. Results from an RCT a priori subgroup analysis.* Australas J Ageing, 2018. **37**(1): p. 54-61.
10. Picorelli, A.M., et al., *Adherence to exercise programs for older people is influenced by program characteristics and personal factors: a systematic review.* J Physiother, 2014. **60**(3): p. 151-6.
11. Knox, C.R., et al., *Treatment compliance and effectiveness of a cognitive behavioural intervention for low back pain: a complier average causal effect approach to the BeST data set.* BMC Musculoskelet Disord, 2014. **15**: p. 17.
12. Taraldsen, K., et al., *Protocol for the PreventIT feasibility randomised controlled trial of a lifestyle-integrated exercise intervention in young older adults.* BMJ Open, 2019. **9**(3): p. e023526.
13. Taraldsen, K., et al., *Digital Technology to Deliver a Lifestyle-Integrated Exercise Intervention in Young Seniors—The PreventIT Feasibility Randomized Controlled Trial.* 2020. **2**(10).
14. Schwenk, M., et al., *The Adapted Lifestyle-Integrated Functional Exercise Program for Preventing Functional Decline in Young Seniors: Development and Initial Evaluation.* Gerontology, 2019. **65**(4): p. 362-374.
15. Boulton, E., et al., *Implementing behaviour change theory and techniques to increase physical activity and prevent functional decline among adults aged 61-70: The PreventIT project.* Prog Cardiovasc Dis, 2019. **62**(2): p. 147-156.
16. Newman-Beinart, N.A., et al., *The development and initial psychometric evaluation of a measure assessing adherence to prescribed exercise: the Exercise Adherence Rating Scale (EARS).* Physiotherapy, 2017. **103**(2): p. 180-185.
17. Fairhall, N., et al., *A multifactorial intervention for frail older people is more than twice as effective among those who are compliant: complier average causal effect analysis of a randomised trial.* J Physiother, 2017. **63**(1): p. 40-44.
18. Jette, A.M., et al., *Late life function and disability instrument: I. Development and evaluation of the disability component.* J Gerontol A Biol Sci Med Sci, 2002. **57**(4): p. M209-16.
19. Paraschiv-Ionescu, A., et al., *Concern about Falling and Complexity of Free-Living Physical Activity Patterns in Well-Functioning Older Adults.* Gerontology, 2018. **64**(6): p. 603-611.
20. Haley, S.M., et al., *Late Life Function and Disability Instrument: II. Development and evaluation of the function component.* J Gerontol A Biol Sci Med Sci, 2002. **57**(4): p. M217-22.

For peer review only - http://bmjopen.bmj.com/site/about/guidelines.xhtml

21. Paraschiv-Ionescu, A., et al., *Barcoding human physical activity to assess chronic pain conditions.* PLoS One, 2012. **7**(2): p. e32239.
22. Paraschiv-Ionescu, A., et al., *Quantifying dimensions of physical behavior in chronic pain conditions.* J Neuroeng Rehabil, 2016. **13**(1): p. 85.
23. Zhang, W., et al., *Complexity of Daily Physical Activity Is More Sensitive Than Conventional Metrics to Assess Functional Change in Younger Older Adults.* Sensors (Basel), 2018. **18**(7).
24. Jonkman, N.H., et al., *Predicting Trajectories of Functional Decline in 60- to 70-Year-Old People.* Gerontology, 2018. **64**(3): p. 212-221.
25. Allison, P.D., *Missing Data*. 2001, Thousand Oaks, CA: Sage University Press.
26. Stuart, E.A., et al., *Estimating intervention effects of prevention programs: accounting for noncompliance.* Prev Sci, 2008. **9**(4): p. 288-98.
27. Herbert, R.D., J. Kasza, and K. Bo, *Analysis of randomised trials with long-term follow-up.* BMC Med Res Methodol, 2018. **18**(1): p. 48.
28. Aiken, L.S. and S.G. West, *Multiple Regression: Testing and Interpreting Interactions*. 1991, Newbury Park: Sage Publications.
29. Peugh, J.L., et al., *Beyond intent to treat (ITT): A complier average causal effect (CACE) estimation primer.* J Sch Psychol, 2017. **60**: p. 7-24.
30. Lamb, S.E., et al., *Development of a common outcome data set for fall injury prevention trials: the Prevention of Falls Network Europe consensus.* J Am Geriatr Soc, 2005. **53**(9): p. 1618-22.
31. French, D.P., et al., *Which behaviour change techniques are most effective at increasing older adults' self-efficacy and physical activity behaviour? A systematic review.* Ann Behav Med, 2014. **48**(2): p. 225-34.
32. Michie, S., et al., *Effective techniques in healthy eating and physical activity interventions: a meta-regression.* Health Psychol, 2009. **28**(6): p. 690-701.
33. Shier, V., E. Trieu, and D.A. Ganz, *Implementing exercise programs to prevent falls: systematic descriptive review.* Inj Epidemiol, 2016. **3**(1): p. 16.
34. Saadat, Z., et al., *Adherence to Antihypertensives in Patients With Comorbid Condition.* Nephrourol Mon, 2015. **7**(4): p. e29863.
35. Marks, R. and J.P. Allegrante, *Chronic osteoarthritis and adherence to exercise: a review of the literature.* J Aging Phys Act, 2005. **13**(4): p. 434-60.
36. Stanton, R. and P. Reaburn, *Exercise and the treatment of depression: a review of the exercise program variables.* J Sci Med Sport, 2014. **17**(2): p. 177-82.
37. Morey, M., et al., *From Supervised to Unsupervised Exercise: Facors associated with Exercise Adherence.* Journal of Aging and Physical Activity, 2003. **11**: p. 351-368.
38. Iliou, M.C., et al., *Cardiac rehabilitation in patients with pacemakers and implantable cardioverter defibrillators.* Monaldi Arch Chest Dis, 2016. **86**(1-2): p. 756.
39. Alkerwi, A., et al., *Adherence to physical activity recommendations and its associated factors: an interregional population-based study.* J Public Health Res, 2015. **4**(1): p. 406.
40. van Gool, C.H., et al., *Effects of exercise adherence on physical function among overweight older adults with knee osteoarthritis.* Arthritis Rheum, 2005. **53**(1): p. 24-32.
41. Roaldsen, K.S., et al., *Task-specific balance training improves self-assessed function in community-dwelling older adults with balance deficits and fear of falling: a randomized controlled trial.* Clin Rehabil, 2014. **28**(12): p. 1189-97.
42. Aguinaga, S., et al., *Home-Based Physical Activity Program Improves Depression and Anxiety in Older Adults.* J Phys Act Health, 2018. **15**(9): p. 692-696.
43. Brach, J.S. and J.M. Vanswearingen, *Interventions to Improve Walking in Older Adults.* Curr Transl Geriatr Exp Gerontol Rep, 2013. **2**(4).
44. Gschwind, Y.J., et al., *The effect of sensor-based exercise at home on functional performance associated with fall risk in older people - a comparison of two exergame interventions.* Eur Rev Aging Phys Act, 2015. **12**: p. 11.

For peer review only - http://bmjopen.bmj.com/site/about/guidelines.xhtml

45. Lacroix, A., et al., *Effects of Supervised vs. Unsupervised Training Programs on Balance and Muscle Strength in Older Adults: A Systematic Review and Meta-Analysis.* Sports Med, 2017. **47**(11): p. 2341-2361.
46. Wu, G., et al., *Comparison of telecommunication, community, and home-based Tai Chi exercise programs on compliance and effectiveness in elders at risk for falls.* Arch Phys Med Rehabil, 2010. **91**(6): p. 849-56.
47. Lord, S.R., et al., *The effect of an individualized fall prevention program on fall risk and falls in older people: a randomized, controlled trial.* J Am Geriatr Soc, 2005. **53**(8): p. 1296-304.
48. Boongird, C., et al., *Effects of a simple home-based exercise program on fall prevention in older adults: A 12-month primary care setting, randomized controlled trial.* Geriatr Gerontol Int, 2017. **17**(11): p. 2157-2163.
49. Sun, X., et al., *Is a subgroup effect believable? Updating criteria to evaluate the credibility of subgroup analyses.* BMJ, 2010. **340**: p. c117.
50. van Overbeeke, E., et al., *Factors and situations influencing the value of patient preference studies along the medical product lifecycle: a literature review.* Drug Discov Today, 2019. **24**(1): p. 57-68.
51. Yardley, L., et al., *Attitudes and beliefs that predict older people's intention to undertake strength and balance training.* The journals of gerontology. Series B, Psychological sciences and social sciences, 2007. **62**(2): p. P119-25.

For peer review only

For peer review only - http://bmjopen.bmj.com/site/about/guidelines.xhtml

Table 1: Demographics

	aLiFE n = 59	eLiFE n = 61
**Age, years (mean, SD)**	66.19 (2.32)	66.43 (2.33)
**Gender, female (n, %)**	30 (50.8%)	33 (54.1%)
**Living alone (n, %)**	21 (36.7%)	18 (29.5%)
**Pain during rest, 0-10, (median, IQR)**	1.0 (1.0-3.0)	1.0 (1.0-3.0)
**Pain during walking, 0-10, (mean, SD)**	2.5 (1.0-4.0)	2.0 (1.0-4.5)
**Falls in past year (n, %)**		
**0**	53 (91.4%)	51 (83.6%)
**1**	4 (6.9%)	10 (16.4%)
**2+**	1 (1.7%)	0 (0)
**Economic satisfaction (n, %)**		
- **Good**	23 (39.7%)	23 (37.7%)
- **Sufficient**	22 (37.9%)	31 (50.8%)
- **Poor/bad**	13 (22.4%)	7 (11.5%)
**Total number comorbidities, (median, IQR)**	2.0 (1.0-4.0)	2.0 (1.0-3.0)
**Total number medication, (median, IQR)**	2.0 (1.0-4.0)	2.0 (1.0- 3.0)
**Confirmed arthritis (n,%)**	19 (32.8%)	18 (29.5%)
**Confirmed cardio-vascular disease (n,%)**	9 (15.5%)	14 (23.0%)
**CES-D score (median, IQR), 0-60**	8.0 (3.0-14.0)	6.5 (3.25-11.0)
**Moderate risk of functional decline (n,%)**	7 (11.9%)	6 (10%)
**LLFDI score (mean, SD)0-100**		
**Functional (overall)**	73.7 (12.9)	73.1(10.6)

For peer review only - http://bmjopen.bmj.com/site/about/guidelines.xhtml

**Lower extremities basic**	85.3 (15.3)	83.6 (12.7)
**Lower extremities advanced**	70.5 (15.7)	71.5 (15.4)
**Upper extremities**	88.0 (12.7)	87.7 (12.1)
**Disability frequency**	52.2 (4.6)	50.5 (4.0)
**Disability limitation (median, IQR) 0-100**	84 (72.6 to 100)	80 (72 to 100)
**Behavioural complexity score (mean, SD)**	0.347 (0.123)	0.374 (0.119)

IQR, interquartile range; SD, standard deviation; CES-D, Center for Epidemiological Studies Depression scale; LLFDI, Late-Life Function and Disability Index.

For peer review only

Table 2: Reported adherence levels

	Monthly adherence reporting				EARS score at six-months follow-up (/24) (mean, SD) (n=106)
**Adherence level**	Full adherence	Partial adherence	Non-adherence	Missing	
Treatment mode					
**All participants**	26	59	21	14	16.02 (5.12)
**eLiFE**	15	27	10	8	16.75 (5.01)
**aLiFE**	11	32	11	*6*	15.29 (5.18)

Missing: 3 participants failed to report their monthly adherence repeatedly, beyond where imputation was possible, further 11 withdrew during follow-up.

EARS, Exercise adherence rating scale; SD, standard deviation.

For peer review only - http://bmjopen.bmj.com/site/about/guidelines.xhtml

Table 3: Determinants of adherence level (measured using the Exercise Adherence Rating Scale).
Multivariate linear regression (n= 120, aLiFE and eLiFE participants)

	Univariate			Multivariate *		
	B (SE)	ß	p-value	B (SE)	ß	p-value
**Sociodemographic**						
**Gender: female**	1.56 (1.01)	0.14	.153			
**Age, (years) §**	-0.12 (0.22)	-0.05	.587			
**Satisfaction with economic situation §**	-0.01 (0.72)	-0.002	.987			
**Living status (alone)~**	2.12 (1.06)	0.20	**.048**			
**Years of education ~**	0.003 (0.11)	0.003	.978			
**Medical history**						
**Number of medication§**	-0.61 (0.21)	-0.29	**.003**	**-0.45 (0.21)**	**-0.21**	**.033**
**Been diagnosed/treated for arthritis ‡**	-2.10 (1.08)	-0.19	.056			
**Been diagnosed/treated for cardiovascular diseases ‡**	-0.47 (1.30)	-0.04	.721			
**Cognition, affect and well-being**						
**MOCA score ~**	0.27 (0.27)	0.10	.337			
**CES-D score ~**	-0.20 (0.08)	-0.25	**.011**	**-0.20 (0.07)**	**-0.25**	**.009**
**Short Form 12 (SF-12) ~**	0.48 (0.17)	0.28	**.006**			
**Physical ability and mobility ‡**						
**Gait speed – usual pace (4m walk)**	5.56 (2.29)	0.24	**.017**			
**Gait speed – usual pace (7m walk)**	4.30 (2.02)	0.21	**.036**			
**Fast gait speed – fast pace (7m walk)**	1.82 (1.02)	0.18	.077			
**CBMS (/96)**	0.11 (0.04)	0.28	**.004**			
**8 Level Balance test (/8)‡**	0.93 (0.52)	0.18	.077			
**Cadence, mean (SD)**	0.07 (0.11)	0.07	.517			
**Complexity, mean (SD)**	-0.10 (4.13)	-0.002	.981			
**Subjective health rating**						
**Pain while walking, VAS~**	-0.31 (0.22)	-0.14	.151			
**Pain during rest, VAS ~**	-0.03 (0.26)	-0.01	.915			
**LLFDI disability frequency (/100)**	0.17 (0.12)	0.14	.150			
**LLFDI disability limitation (/100)**	0.08 (0.04)	0.21	**.032**			

For peer review only

For peer review only - http://bmjopen.bmj.com/site/about/guidelines.xhtml

**LLFDI function total (/100)**	0.10 (0.04)	0.24	**.002**			
**LLFDI function UE**	0.08 (0.03)	0.19	**.015**			
**LLFDI function BLE**	0.09 (0.04)	0.25	**.011**			
**LLFDI function ALE**	0.10 (0.03)	0.28	**.004**			
**<u>Other potential adherence mediators</u>**						
**Group allocation aLiFE vs eLiFE**	-1.45 (1.01)	-0.14	.153			
**Risk screening moderate category**	-0.08 (0.02)	-0.03	**.006**	**-0.06 (0.03)**	**-0.21**	**.035**

~ Factors which influence adherence; * only variables retained in the final model presented; ǂ Strength and balance deficits known to influence adherence; § Know risk factors for functional decline; SD, standard deviation; MOCA, Montreal Cognitive Assessment; CES-D, Center for Epidemiological Studies Depression scale; CBMS, community balance and mobility scale; VAS, visual analogue scale; LLFDI, Late-Life Function and Disability Index; UE, upper extremity; BLE, basic lower extremity; ALE, advanced lower extremity; CG, control group;

For peer review only

For peer review only - http://bmjopen.bmj.com/site/about/guidelines.xhtml

Table 4: **Estimated association of adherence and group allocation at follow-up** (aLiFE and eLiFE; n=120)

	Unstandardized B	Coefficients Std. Error	Stnβ	95% CI	p Value
**<u>Primary outcome measures</u>**					
**<u>Late Life Function and Disability Index (LLFDI)</u>**					
**LLFDI disability frequency**					
**Baseline score**	.677	.065	.716	.549 to .80	.000
**Group allocation**	.872	.553	.105	-.226 to 1.970	.118
**Predicted adherence level**	.080	.127	.042	-.173 to .333	.533
**LLFDI disability limitation (/100)**					
**Baseline score**	.663	.081	.621	.502 to .824	.000
**Group allocation**	-.487	1.968	-.017	-4.390 to 3.417	.805
**Predicted adherence level**	.663	.081	.621	.346 to 2.393	**.009**
**LLFDI function total components**					
**Baseline score**	.779	.064	.760	.652 to .906	.000
**Group allocation**	-1.354	1.260	-.057	-3.854 to 1.146	.285
**Predicted adherence level**	.797	.344	.144	.114 to 1.480	**.023**
**LLFDI UE**					
**Baseline score**	.624	.074	.623	.478 to .771	.000
**Group allocation**	-.667	1.694	-.027	-4.028 to 2.693	.694
**Predicted adherence level**	1.113	.418	.197	.285 to 1.942	**.009**
**LLFDI BLE**					
**Baseline score**	.632	.076	.631	.481 to .784	.000
**Group allocation**	-1.994	1.788	-.072	-5.540 to 1.552	.267
**Predicted adherence level**	1.304	.486	.204	.339 to 2.268	**.009**
**LLFDI ALE**					
**Baseline score**	.868	.066	.780	.736 to .999	.000
**Group allocation**	-1.934	1.763	-.057	-5.431 to 1.562	.275
**Predicted adherence level**	1.000	.467	.127	.074 to 1.926	**.035**

For peer review only

For peer review only - http://bmjopen.bmj.com/site/about/guidelines.xhtml

**Behavioural complexity**					
**Complexity, mean**					
**Baseline score**	.178	.076	.235	.028 to .329	.021
**Group allocation**	-.012	.019	-.063	-.049 to .025	.525
**Predicted adherence level**	.008	.004	.185	-.001 to .017	.068
**Complexity, median (IQR)**					
**Baseline score**	.209	.076	.270	.057 to .360	.008
**Group allocation**	-.007	.019	-.035	-.045 to .031	.726
**Predicted adherence level**	.008	.004	.186	-.045 to .017	.063
**Secondary outcome measures**					
**Cognition, affect and well-being**					
**MOCA score**					
**Baseline score**	.590	.090	.558	.411 to .769	.000
**Group allocation**	.190	.328	.049	-.461 to .842	.563
**Predicted adherence level**	-.023	.077	-.026	-.176 to .129	.762
**CES-D score**					
**Baseline score**	.426	.087	.444	.253 to .598	.000
**Group allocation**	-.586	.903	-.048	-2.377 to 1.205	.518
**Predicted adherence level**	-.889	.257	-.313	-1.399 to -.380	**.001**
**Short Form 12 (SF-12)**					
**Baseline score**	.500	.102	.467	.297 to .703	.000
**Group allocation**	-.990	.517	-.158	-2.017 to .037	.059
**Predicated adherence level**	.231	.138	.160	-.042 to .505	.097
**Physical ability and mobility** ‡					
**Usual gait speed (7m walk)**					
**Baseline score**	.621	.057	.717	.507 to .735	.000
**Group allocation**	.041	.027	.097	-.012 to .095	.128
**Predicted adherence level**	.014	.007	.141	.001 to .027	**.036**
**CBMS (/96)**					
**Baseline score**	.847	.073	.796	.702 to .992	.000
**Group allocation**	1.039	1.599	.038	-2.134 to 4.212	.517

For peer review only

For peer review only - http://bmjopen.bmj.com/site/about/guidelines.xhtml

**Predicated adherence level**	.206	.431	.033	-.648 to 1.061	.633
**8 Level Balance test**					
**Baseline score**	.467	.116	.396	.237 to .697	.000
**Group allocation**	.254	.213	.111	-.168 to .677	.235
**Predicted adherence level**	.047	.051	.089	-.053 to .147	.356

MOCA, Montreal Cognitive Assessment; CES-D, Center for Epidemiological Studies Depression scale; CBMS, community balance and mobility scale; VAS, visual analogue scale; LLFDI, Late-Life Function and Disability Index; UE, upper extremity; BLE, basic lower extremity; ALE, advanced lower extremity;

For peer review only

Reporting checklist for randomised trial.

Based on the CONSORT guidelines.

Instructions to authors

Complete this checklist by entering the page numbers from your manuscript where readers will find each of the items listed below.

Your article may not currently address all the items on the checklist. Please modify your text to include the missing information. If you are certain that an item does not apply, please write "n/a" and provide a short explanation.

Upload your completed checklist as an extra file when you submit to a journal.

In your methods section, say that you used the CONSORTreporting guidelines, and cite them as:

Schulz KF, Altman DG, Moher D, for the CONSORT Group. CONSORT 2010 Statement: updated guidelines for reporting parallel group randomised trials

		Reporting Item	Page Number
**Title and Abstract**			
Title	#1a	Identification as a randomized trial in the title.	1
Abstract	#1b	Structured summary of trial design, methods, results, and conclusions	2
**Introduction**			

For peer review only

Background and objectives	#2a	Scientific background and explanation of rationale	4
Background and objectives	#2b	Specific objectives or hypothesis	5
**Methods**			
Trial design	#3a	Description of trial design (such as parallel, factorial) including allocation ratio.	5
Trial design	#3b	Important changes to methods after trial commencement (such as eligibility criteria), with reasons	n/a
Participants	#4a	Eligibility criteria for participants	6
Participants	#4b	Settings and locations where the data were collected	6
Interventions	#5	The experimental and control interventions for each group with sufficient details to allow replication, including how and when they were actually administered	6
Outcomes	#6a	Completely defined prespecified primary and secondary outcome measures, including how and when they were assessed	7
Sample size	#7a	How sample size was determined.	6
Sample size	#7b	When applicable, explanation of any interim analyses and stopping guidelines	n/a

For peer review only

Randomization - Sequence generation	#8a	Method used to generate the random allocation sequence.	
6			
Randomization - Sequence generation	#8b	Type of randomization; details of any restriction (such as blocking and block size)	
6			
Randomization - Allocation concealment mechanism	#9	Mechanism used to implement the random allocation sequence (such as sequentially numbered containers), describing any steps taken to conceal the sequence until interventions were assigned	6
Randomization - Implementation	#10	Who generated the allocation sequence, who enrolled participants, and who assigned participants to interventions	6
Blinding	#11a	If done, who was blinded after assignment to interventions (for example, participants, care providers, those assessing outcomes) and how.	6
Blinding	#11b	If relevant, description of the similarity of interventions	n/a
Statistical methods	#12a	Statistical methods used to compare groups for primary and secondary outcomes	10
Statistical methods	#12b	Methods for additional analyses, such as subgroup analyses and adjusted analyses	10

For peer review only

Outcomes	#6b	Any changes to trial outcomes after the trial commenced, with reasons	n/a
**Results**			
Participant flow diagram (strongly recommended)	#13a	For each group, the numbers of participants who were randomly assigned, received intended treatment, and were analysed for the primary outcome	11
Participant flow	#13b	For each group, losses and exclusions after randomization, together with reason	n/a
Recruitment	#14a	Dates defining the periods of recruitment and follow-up	n/a
Recruitment	#14b	Why the trial ended or was stopped	n/a
Baseline data	#15	A table showing baseline demographic and clinical characteristics for each group	24
Numbers analysed	#16	For each group, number of participants (denominator) included in each analysis and whether the analysis was by original assigned groups	11, 24
Outcomes and estimation	#17a	For each primary and secondary outcome, results for each group, and the estimated effect size and its precision (such as 95% confidence interval)	26
Outcomes and estimation	#17b	For binary outcomes, presentation of both absolute and relative effect sizes is recommended	26

For peer review only

Ancillary analyses	#18	Results of any other analyses performed, including subgroup analyses and adjusted analyses, distinguishing pre-specified from exploratory	26, 28
Harms	#19	All important harms or unintended effects in each group (For specific guidance see CONSORT for harms)	n/a
**Discussion**			
Limitations	#20	Trial limitations, addressing sources of potential bias, imprecision, and, if relevant, multiplicity of analyses	17
Interpretation	#22	Interpretation consistent with results, balancing benefits and harms, and considering other relevant evidence	16
Registration	#23	Registration number and name of trial registry	6
**Other Information**			
Protocol	#24	Where the full trial protocol can be accessed, if available	6
Funding	#25	Sources of funding and other support (such as supply of drugs), role of funders	19

The CONSORT checklist is distributed under the terms of the Creative Commons Attribution License CC-BY. This checklist was completed on 06. June 2021 using https://www.goodreports.org/, a tool made by the EQUATOR Network in collaboration with Penelope.ai

For peer review only

BMJ Open

Impact of adherence to a Lifestyle-integrated Programme on physical function and behavioural complexity in young older adults at risk of functional decline: a multicentre RCT secondary analysis.

Journal:	*BMJ Open*
Manuscript ID	bmjopen-2021-054229.R1
Article Type:	Original research
Date Submitted by the Author:	29-Nov-2021
Complete List of Authors:	Mikolaizak, A. Stefanie; Robert-Bosch-Krankenhaus GmbH, Department of Clinical Gerontology<br>Taraldsen, Kristin; Oslo Metropolitan University, Department of Physiotherapy; Norwegian University of Science and Technology, Department of Neuromedicine and Movement Science<br>Boulton, Elisabeth; University of Manchester, School of Health Sciences; Manchester Academic Health Science Centre,<br>Gordt, Katharina; Robert-Bosch-Krankenhaus GmbH,<br>Maier, Andrea; The University of Melbourne, Department of Human Movement Sciences<br>Mellone, Sabato; University of Bologna, Department of Electrical, Electronic and Information Engineering «Guglielmo Marconi»<br>Hawley-Hague, Helen; University of Manchester, School of Health Sciences<br>Aminian, Kamiar; Ecole Polytechnique Federale de Lausanne<br>Chiari, Lorenzo; University of Bologna, Department of Electrical, Electronic and Information Engineering «Guglielmo Marconi»<br>Paraschiv-Ionescu, Anisoara; Ecole Polytechnique Federale de Lausanne<br>Pijnappels, Mirjam; Vrije Universiteit Amsterdam, Department of Human Movement Sciences<br>Todd, Chris; University of Manchester, School of Health Sciences<br>Vereijken, Beatrix; Norwegian University of Science and Technology (NTNU), Department of Neuromedicine and Movement Science<br>Helbostad, Jorunn; Norwegian University of Science and Technology<br>Becker, Clemens; RobertBoschKrankenhaus, Geriatric Medicine
**Primary Subject Heading**:	Geriatric medicine
Secondary Subject Heading:	Public health
Keywords:	Telemedicine < BIOTECHNOLOGY & BIOINFORMATICS, GERIATRIC MEDICINE, PUBLIC HEALTH, SPORTS MEDICINE

I, the Submitting Author has the right to grant and does grant on behalf of all authors of the Work (as defined in the below author licence), an exclusive licence and/or a non-exclusive licence for contributions from authors who are: i) UK Crown employees; ii) where BMJ has agreed a CC-BY licence shall apply, and/or iii) in accordance with the terms applicable for US Federal Government officers or employees acting as part of their official duties; on a worldwide, perpetual, irrevocable, royalty-free basis to BMJ Publishing Group Ltd ("BMJ") its licensees and where the relevant Journal is co-owned by BMJ to the co-owners of the Journal, to publish the Work in this journal and any other BMJ products and to exploit all rights, as set out in our licence.

The Submitting Author accepts and understands that any supply made under these terms is made by BMJ to the Submitting Author unless you are acting as an employee on behalf of your employer or a postgraduate student of an affiliated institution which is paying any applicable article publishing charge ("APC") for Open Access articles. Where the Submitting Author wishes to make the Work available on an Open Access basis (and intends to pay the relevant APC), the terms of reuse of such Open Access shall be governed by a Creative Commons licence – details of these licences and which Creative Commons licence will apply to this Work are set out in our licence referred to above.

Other than as permitted in any relevant BMJ Author's Self Archiving Policies, I confirm this Work has not been accepted for publication elsewhere, is not being considered for publication elsewhere and does not duplicate material already published. I confirm all authors consent to publication of this Work and authorise the granting of this licence.

For peer review only

Impact of adherence to a Lifestyle-integrated Programme on physical function and behavioural complexity in young older adults at risk of functional decline: a multicentre RCT secondary analysis.

Mikolaizak AS, PhD[1], Taraldsen K, PhD[2,3], Boulton E, PhD[4,5] , Gordt K, PhD[1], Maier AB, MD,PhD[6,7], Mellone S[8], PhD, Hawley-Hague H,PhD[4,5], Aminian K, PhD[9], Chiari L,PhD[8], Paraschiv-Ionescu A,PhD[9], Pijnappels M,PhD[6], Todd C, PhD[4,5,10], Vereijken B, PhD[3], Helbostad JL,PhD[3], Becker C,MD,PhD[1]

[1] Department of Clinical Gerontology, Robert Bosch Krankenhaus, Stuttgart, Germany; Stefanie.mikolaizak@rbk.de; katharina.gordt@issw.uni-heidelberg.de; Clemens.Becker@rbk.de

[2] Department of Physiotherapy, Faculty of Health Sciences, Oslo Metropolitan University (OsloMet), Oslo, Norway; kristin.taraldsen@oslomet.no

[3] Department of Neuromedicine and Movement Science, Norwegian University of Science and Technology (NTNU), Trondheim, Norway; beatrix.vereijken@ntnu.no; jorunn.helbostad@ntnu.no

[4] School of Health Science, Faculty of Biology, Medicine and Health, University of Manchester, Manchester, UK; elisabeth.boulton@manchester.ac.uk; helen.hawley-hague@manchester.ac.uk; Chris.Todd@manchester.ac.uk

[5] Manchester Academic Health Science Centre, Manchester, UK

[6]Department of Human Movement Sciences, @AgeAmsterdam, Amsterdam Movement Sciences, Vrije Universiteit, Amsterdam, The Netherlands; m.pijnappels@vu.nl

[7]Department of Medicine and Aged Care, @AgeMelbourne, The Royal Melbourne Hospital, The University of Melbourne, Parkville, Vic., Australia; Andrea.Maier@mh.org.au

For peer review only - http://bmjopen.bmj.com/site/about/guidelines.xhtml

[8] Department of Electrical, Electronic and Information Engineering «Guglielmo Marconi», University of Bologna, Bologna, Italy; sabato.mellone@unibo.it; lorenzo.chiari@unibo.it

[9] Laboratory of Movement Analysis and Measurement, Ecole Polytechnique Federale de Lausanne, Lausanne, Switzerland; kamiar.aminian@epfl.ch; anisoara.ionescu@epfl.ch

[10] Manchester University NHS Foundation Trust, Manchester, UK.

Corresponding Author:

A. Stefanie Mikolaizak

Department of Clinical Gerontology, Robert Bosch Krankenhaus,

Stuttgart, Germany

Email: Stefanie.mikolaizak@rbk.de

Word count main text: 4025

Data elements: 4 tables

For peer review only

Abstract

Context: Long-term adherence to physical activity interventions is challenging. The Lifestyle-integrated Functional Exercise (LiFE) programme was adapted (aLiFE) to include more challenging activities and a behavioural change framework, and then enhanced (eLiFE) to be delivered using smartphones and smartwatches.

Objectives: 1) compare adherence measures; 2) identify determinants of adherence, and 3) assess the impact on outcome measures of a lifestyle-integrated programme.

Design, setting, and participants: A multicentre, feasibility randomised controlled trial, in participants aged 61-70 years conducted in three European cities.

Interventions: Six months trainer supported aLiFE or eLiFE compared against a control group, who received written physical activity advice.

Outcome measures: Self-reporting adherence monthly using a single question, and after six months intervention using the Exercise Adherence Rating Scale (EARS; Score range 6-24). Treatment outcomes included function and disability scores (measured using the Late-Life Function and disability Index) and sensor-derived physical behaviour complexity measure. Determinants of adherence (EARS score) were identified using linear multivariate analysis. Linear regression estimated the association of adherence on treatment outcome.

Results: We included 120 participants randomised to the intervention groups (aLiFE/eLiFE) (66.3±2.3 years, 53% females). The 106 participants re-assessed after six months had mean EARS score of 16.0±5.1. Better adherence was associated with lower: number of medications taken, depression and risk of functional decline. We estimated adherence to significantly increase basic lower extremity function by 1.3 points ($p<.0001$), advanced lower extremity function by 1.0 points ($p<.0001$) and behavioural complexity by .008 per one point higher EARS score ($F(3,91)=3.55$, $p=.017$); regardless of group allocation.

For peer review only - http://bmjopen.bmj.com/site/about/guidelines.xhtml

Conclusion: Physical activity adherence was associated with better lower extremity function and physical behavioural complexity. Barriers to adherence should be addressed pre-intervention, to enhance intervention efficacy. Further research is needed to unravel the impact of behaviour change techniques embedded into technology-delivered activity interventions on adherence.

Trial registration: NCT 03065088

For peer review only

Key words: physical activity, mobile health application, intervention, randomised controlled trial, behavioural change

Strengths and limitations of the study

- Our study includes data from a three-arm, three site, international research study, thus eliminating cultural bias towards the intervention.
- Adherence was defined *a-priori* to better capture intervention impact.
- A complier average causal effect analysis was not possible, therefore association between adherence and outcome could only be established, not causation.
- A comprehensive collection of adherence data was included, both as a single question reported monthly throughout the study period as well as a questionnaire at the assessments at 6 and 12 months.
- The monthly measurement of adherence may have acted as a prompt to complete planned activities, although this would be common across all intervention groups and participants.

For peer review only - http://bmjopen.bmj.com/site/about/guidelines.xhtml

Background

Exercise interventions conducted as randomised controlled trials are considered the gold standard for allowing causal inference to be made regarding the efficacy of a treatment. However, the reality is that the participants' adherence to treatment is typically imperfect. The actual intervention effect can only be evaluated accurately if adherence levels are high. Thus, conclusions on treatment effects of an exercise intervention will only be valid if the adherence levels to the protocol are evaluated [1, 2], with several studies reporting poor adherence rates among the study population to mediate the lack of treatment effect [3]. Further disentangling treatment (e.g. type of delivery mode) and exercise effect (e.g. type of exercise) on outcomes is essential when promoting benefits achieved through exercise interventions.

The World Health Organization defines adherence as the '*extent to which a person's behaviour corresponds with the recommendations from a healthcare provider*' [4] and it involves an active choice from the participant to follow through and to take responsibility for their well-being. In pharmaceutical trials, the intervention is 'unidimensional' and is based on patients taking the required dose, which can be monitored using bioanalytical methods. Interventions involving exercise and physical activity are more complex and 'multi-dimensional', as, for example, variance in daily form/fitness or required training effect influence the intensity required to maintain a specific training dose.

To date, there is no consensus regarding which adherence measurement tool should be used, nor when and how adherence should be measured [5, 6]. Besides the difficulty of recording adherence rates, achieving sufficiently high adherence is challenging. High adherence is frequently documented in the early stages of an exercise intervention, regardless of delivery mode and exact definition used [2, 3]. In contrast, long-term intervention adherence can be as low as 30% [2], with activity levels reverting to pre-intervention levels following the

For peer review only - http://bmjopen.bmj.com/site/about/guidelines.xhtml

intervention period. Integrating tailored activities into daily life help achieve longer-term adherence levels than a structured programme, where exercise is segregated from daily routines [7]. While this delivery method is gaining in popularity[8], there is limited knowledge on how to assess adherence to a lifestyle-integrated activity programme. Furthermore, a recent systematic review showed that interventions that used telecommunication, and those offering lifestyle-integrated exercise interventions, appeared most promising for maintaining adherence[3]. Despite this, interventions that specifically targeted adherence did not affect balance or gait parameters.

Adherence to exercise appears to be influenced by socioeconomic status, health, physical function, motivation and other psychological variables [9, 10]. Understanding which factors influence young older adults (60-70 years old) to adhere to lifestyle-integrated exercises is essential for future intervention success [3]. Modifiable factors that influence adherence need to be identified, ideally pre-treatment, in order to influence treatment effect.

This paper reports *a priori* secondary analysis of the PreventIT randomised controlled feasibility trial data to investigate factors influencing adherence to a personalised lifestyle-integrated exercise intervention offered to young older adults, with the intention to prevent accelerated functional decline. Here, adherence is defined as participants completing all their planned lifestyle-integrated activities into daily life, as described below. Further, adherence was hypothesised to be a treatment-effect modifier which needs to be analysed within the intervention groups. The current *a-priori* analysis based on adherence levels is warranted. It may have greater clinical relevance, be more indicative of the provided treatment effect [11], aid future improvements of the intervention programme, and guide implementation into practice.

For peer review only - http://bmjopen.bmj.com/site/about/guidelines.xhtml

Aims

The aims of this analysis are to: 1) assess whether measuring adherence monthly prospectively, and retrospectively every six months, yields the same results; 2) identify baseline characteristics that determine adherence levels, and 3) estimate the association of adherence to a lifestyle-integrated intervention on primary and secondary outcome measures (functional ability and behavioural complexity) in young older adults.

METHODS

Design

This *a-priori* analysis is part of a large-scale multicentre RCT - PreventIT (Clinical trial NCT 03065088), analysing the impact of a 6-month active intervention period within a 12- month trial. PreventIT, a personalised behavioural change exercise intervention for young older adults, was developed to prevent accelerated functional decline. The detailed protocol [12] and results of the feasibility analysis, including an estimate of change for the primary outcome measures have been published elsewhere[13]. Ethical approval was obtained the relevant ethical committees of the clinical sites (Norway (REK midt, 2016/1891), Stuttgart (registration number 770/2016BO1), and Amsterdam [METc VUmc registration number 2016.539 (NL59977.029.16)]. The patients/participants provided their written informed consent to participate in this study.

Participants

The trial sample for this pre-planned analysis includes the intervention group participants from the two treatment modes (n=120). Participants were invited to participate if they were aged 61-70 years, retired or working part-time, community dwelling and able to walk 500 meters without a walking aid. Exclusion criteria included participation in an exercise class (>1/week) or undertaking moderate-intensity physical activity (≥150 min/week).

Intervention

For peer review only - http://bmjopen.bmj.com/site/about/guidelines.xhtml

Participants received tailored exercises at an individual level and learned to integrate these into everyday situations. The same activity framework was applied in both intervention arms, and was delivered in two different modes: via paper-pen manuals (aLiFE) or via a mobile health application system (eLiFE). Participants received between one and six home visits, depending on group allocation, from an exercise physiologist/scientist or physiotherapist across six months during which the exercise program was taught and supported, with the intention for participants to learn to progress their exercises independently The programme consisted of strategies to a) improve balance; b) increase muscle strength; and c) reduce sedentariness and increase physical activity (PA). The concept, based on the LiFE programme [7], has been adapted to young older adults to be more challenging [14], and is underpinned by a behavioural change framework to support older adults to form long-term physical activity habits. PreventIT has taken the original LiFE concept and further developed the behaviour change elements, explicitly mapping them to Social Cognitive Theory, Habit Formation Theory, and 30 Behaviour Change Techniques (BCTs)[15] Goal setting, planning, prompts, and real-time feedback are used to deliver a person-centred experience. The full interventions details have been published [12]

Participants received their randomly allocated intervention during the first six months (active period) of the programme and were then encouraged to keep up their programme (passive period) independently for another six months. Here, an analysis of the first 6-month active intervention period is presented.

For context, control group participants received general WHO advice on the benefits of physical activity but no tailored exercise program. Therefore an exercise adherence analysis was not relevant for this group.

Exercise adherence was encouraged in general to all participants and participants were provided with adherence logs to document their activity. No incentives or additional adherence promotion was provided to the participants within the PreventIT trial.

For peer review only - http://bmjopen.bmj.com/site/about/guidelines.xhtml

Outcome measures

Participants underwent a comprehensive assessment by an assessor blinded to group allocation at baseline, after a 6-month active, and a further 6-month passive intervention period.

<u>Adherence measures</u>

Adherence was collected using two methods in this trial.

Exercise Adherence Reporting Scale at post-test and follow-up

The Exercise Adherence Reporting Scale (EARS) [16] was completed by participants during follow-up as an objective, reliable, and validated self-report measure of adherence to planned (exercise) activities. EARS consists of 16 questions, each answered on a 5-point Likert-scale (scored 0-4), identifying how strongly the participant agrees/disagrees with each statement. Six questions capture the level of adherence, while 10 questions address facilitators or barriers of adherence. A higher score indicates better adherence; however, a pre-defined cut-off point for adequate adherence does not exist. Adherence is frequently dichotomised, based on an arbitrary cut-off, however, adherence with an ongoing complex treatment is variable, and analysing adherence as a continuous or ordinal variable is warranted [17].

Monthly reports of adherence

Every month, participants were asked to report, on a single-page questionnaire by ticking one of seven options, whether they had completed their planned activities 'yes- more than planned', 'yes -as much as planned', 'yes – but not as much as planned' or 'No - not at all' because i) 'they did not feel well', ii) 'they forgot', iii) 'they did not like the activities' or iv) 'they did not have time'. Response to the single question was possible via email or postcard where each month of reporting was summarized as full adherence (responded positively, and "more than or

For peer review only - http://bmjopen.bmj.com/site/about/guidelines.xhtml

as much as planned"), partial adherence (responded positively, and "but not as much as planned"), or non-adherence (not at all, regardless of reason). Data from participants who provided four or more responses during the six-month intervention and follow-up period were included in the analysis. The validity of this novel method of assessing adherence was tested within the PreventIT feasibility trial [13].

Treatment outcome measures

The trial included two primary outcome measures [12]: subjective health rating using the Late-Life Function and Disability Index (LLFDI) [18], and physical activity measure using a behavioural complexity metric [19].

The LLFDI is a comprehensive questionnaire assessing function (ability to perform specific activities of daily living) and disability (inability to take part in major life tasks and social roles) for use in community-dwelling older adults [18, 20].

The behavioural complexity metric was derived from a 7-day consecutive physical activity monitoring period, and assessed in the domains of daily physical activity, and social participation. A 3-Axis Logging Accelerometer (https://axivity.com/product/ax3) attached to participants' lower back (using adhesive tape) represent physical behaviours as time series embedding activity characteristics (i.e., type, duration, intensity, and dynamics of transitions between activities) [19, 21] . The concept of behavioural complexity, initially developed to assess physical behaviour in chronic pain patients [21, 22], was further developed in the PreventIT trial using novel computational complexity methods, and the relationship of complexity metrics with additional clinical scores/outcomes, such as fear of falling, and functional balance and mobility performance pre- and post-intervention [19, 23]. The value of behavioural complexity metric ranges from a minimal value of 0.1 for very impaired frail elderly to a maximal value of around 0.7 for highly active subjects. For context, published data for well-functioning older adults indicated a value of 0.40±0.07 (mean±std) for fully confident

For peer review only - http://bmjopen.bmj.com/site/about/guidelines.xhtml

older adults without a fear of falling, and 0.30±0.06 for those active but less confident in their ability [19].

Additional outcome measures included general health and function, medication use, neuropsychological assessment, physical function and risk of functional decline (Table 1). The PreventIT risk screening tool was used to identify and provide a participant's risk estimate (low, medium, or high risk) for the functional decline over the next nine years [24].

Determinants of adherence

Key factors which were anticipated to positively or negatively be associated with participants' adherence were selected from the outcome measures obtained during the PreventIT baseline assessment [13]. Determinates were chosen if they were (i) known functional decline risk factors (e.g. age, or number of medications); (ii) strength or balance deficits, identified during assessment; (iii) potentially associated with adherence (e.g., pain when walking, cognitive impairment) [12].

Statistical analysis

Descriptive statistics were used to summarise demographic data. Baseline comparisons depending on adherence levels were performed using parametric and nonparametric tests as appropriate. Initially, the association between adherence, as reported monthly during the active intervention period (0-6 months post randomisation), and EARS sum score of Question 1 to Question 6, reported at six months post-randomisation, was assessed using Pearson's correlation. In further analyses the continuous EARS score was used.

The modal response was used as an imputation method to account for missing monthly reporting data, which was missing completely at random, allowing 106 participants (53 from each treatment arm) to be included in univariate/multivariate analysis to reduce bias [25].

For peer review only - http://bmjopen.bmj.com/site/about/guidelines.xhtml

Univariate associations between potential determinants and adherence (EARS score) were assessed using logistic regression. To ensure the collinearity assumption was met, potential variables were required to be measuring/assessing uniquely different outcomes and a restricted number of variables was selected determined by the number of available observations to reduce the risk of type I error. Variables identified during univariate regression ($p<0.05$) were subsequently entered into a backward (elimination) multivariate linear regression model to determine the best set of explanatory variables.

In lieu of a suitable complier average causal effect (CACE) analysis, which was not possible given the intention-to-treat analysis showed no difference in effect between groups, multiple linear regression was performed while still honouring the CACE assumptions [26]. Multiple linear regression was used to estimate the association of predicted adherence on primary and secondary outcome measures at six months follow-up, using group allocation (aLiFE and eLiFE), baseline values, and predicted adherence levels as independent variables. Participants' predicted adherence levels (EARS score) were derived using multiple linear regression results [27, 28]. Predicted adherence levels were centred [29] and the interaction association between group allocation and adherence level is presented only when significant. Main associations of baseline values, group allocation and adherence levels are presented. Analyses were conducted using SPSS version 25.0 (SPSS Corp).

RESULTS

Fifty-nine participants were randomised to receive the traditional paper-pen based (aLiFE) and 61 the technology supported (eLiFE) programme. The six months follow-up was completed by 53 (89.8%) aLIFE and 53 (86.9%) eLIFE participants. Participant characteristics are presented in Table 1. Mean age of participants was 66 years (SD: 2.3) and an equal number of male and

For peer review only - http://bmjopen.bmj.com/site/about/guidelines.xhtml

female were enrolled. Most participants had not experienced a fall in the past year, were taking two medications daily and were overall in good health.

[INSERT TABLE 1 HERE]

Monthly and six-monthly adherence measurement

During the six months follow-up period, which also included monthly follow-up, 26 (24.5%) participants reported fully adhering to their planned activity, whilst 21 (19.8%) participants reported non-adherence (Table 2). The remainder (59, 55.7%) were classified as partial adherers. During the six-months follow-up assessment, the mean EARS score was 16.02 (SD: 5.12). These results showed a large, positive, and significant association between the monthly adherence reporting and the EARS questionnaire score at six months ($r(102)= .618$, $p<0.001$). The mean EARS scores increased with increased adherence, as measured on a monthly basis, with non-adherers scoring as low as 4 out of 24 points and full adherers having a mean of 20 out of 24 on their EARS score.

[INSERT TABLE 2 HERE]

Determinants of adherence

Univariate analyses identified nine baseline variables to be associated with intervention adherence using EARS (Table 3), of which three variables were retained in the multivariate linear regression model: Greater number of medication (Beta = -.21, p = 003), CES-D score (Beta = -.25, p = .009), and the risk of functional decline score (Beta = -.21, p = .35) all significantly influenced poorer adherence levels.

[INSERT TABLE 3 HERE]

For peer review only - http://bmjopen.bmj.com/site/about/guidelines.xhtml

Estimated association of adherence on outcome measures

After controlling for group allocation, predicted adherence level was positively associated with improved physical function, behavioural complexity and well-being (Table 4) at follow-up. Group allocation was not significantly associated with an improvement in any primary or secondary outcome measure.

Treatment outcomes

Higher predicted adherence levels were significantly associated with improved LLFDI scores. In summary, one EARS point higher resulted in 0.67 point improvement on self-reported disability limitation (higher score indicates less perceived limitation) ($F(3,102)=42.50$, $p<.001$). An improvement in all LLFDI function subcomponents was significantly associated with improved adherence. For each EARS point higher, upper extremity (UE), basic lower extremity (BLE), and advanced lower extremity (ALE) function improved by 1.11 points ($F(3,102)=35.90$, $p<.001$), 1.30 points ($F(3,102)=46.60$, $p<.001$), and 1.0 points ($F(3,102)=90.07$, $p<.001$), respectively.

Behavioural complexity increased by 0.08 per point increase in EARS score; however, this change was not significant.

CES-D scores was reduced by 0.89 points ($F(3,102)=28.53$, $p<.001$), while walking speed increased by 0.014m/s ($F(3,102)=50.28$, $p<.001$) per point increase in EARS score.

[INSERT TABLE 4 HERE]

Discussion

For peer review only - http://bmjopen.bmj.com/site/about/guidelines.xhtml

In this analysis we have demonstrated that within this cohort, monthly reporting of adherence (prospective) correlated strongly with a single retrospective report of adherence (EARS) after a six-month intervention period. We identified three main factors that influenced young older adults' adherence to their planned activity: the number of medications they were taking, their level of depression, and their risk score of accelerated functional decline. Higher predicted adherence levels had a significant and clinically meaningful benefit on self-reported function (LLFDI) and greater behavioural complexity. Further, predicted adherence was significantly associated with improved gait speed and lower depression scores. To our knowledge this is the first analysis that examines the association of adherence to a lifestyle-integrated exercise programme with the intention to prevent functional decline in young older adults.

Measuring adherence

To date, there is no consensus on how adherence should be measured. Of the existing approaches [6] a non-standardised, arbitrary binary cut-off is commonly used to categorise participants into 'adherers' and 'non-adherers'. This approach has two main flaws. First, it fails to acknowledge that adherence is a complex construct of continuous quantity [17]. Second, dichotomising participants based on an arbitrary cut-off assumes that partial adherence has a nil treatment effect, when in fact some adherence may affect the dependent outcome measure [30]. Even not completing activities as often or as intended, any activity is still breaking up sedentary behaviour. Within this trial, we used monthly reporting of adherence, building on the gold-standard for prospective fall data collection [31], to eliminate recall bias due to delayed reporting. Further, we used the validated EARS questionnaire after the 6-month active intervention period. We found strong and highly significant correlation between the monthly (prospective) and the bi-annual (retrospective) responses, indicating that a single measurement time-point could suffice during the active intervention period. Further, there is a clear differentiation in mean EARS score and minimum scores amongst the three different adherence

For peer review only - http://bmjopen.bmj.com/site/about/guidelines.xhtml

levels, supporting the opinion that six-monthly EARS assessment can capture details of individuals' adherence levels without requiring monthly questionnaires and the associated potential reporting bias. Ultimately, the method chosen to measure adherence should be determined by its purpose [6].

Monthly adherence reporting allows for tracking the change in adherence over time. From an intervention perspective, this could be used as a facilitator to identify adherence problems early on and address potential barriers in a timely fashion. However, monthly adherence reports are also a form of intervention in themselves, as they acted as reminders to complete the planned activities [13]. Regular prompting has been shown to improve adherence [32], and prompts in themselves are part of the BCT taxonomy [33]. Further, frequent reporting could also be considered burdensome on the participant, and a single retrospective questionnaire may be preferred. Within our young older population using EARS every six months seems sufficient to measure adherence accurately when the purpose of adherence documentation is to obtain an average score per participant, following a six-month intervention period.

Determinants of adherence

Adherence to the lifestyle-integrated activity programme varied among participants, with participants reporting on average 16 out of 24 points on EARS, indicating agreeable and positive intent to adhere. Such variability in adherence has repeatedly been reported for exercise interventions involving older adults [2] and is slightly higher than reported elsewhere [16].

Previously, it has been demonstrated that external factors impact adherence behaviour such as location, frequency of exercising, level of supervision and delivery mode [34]. Also, there are numerous patient-specific, internal factors that directly or indirectly appear important for adherence behaviour [9, 10]. Within our study population, the number of medications used, depression level and risk of accelerated functional decline were negatively associated with adherence. Increased medication use could be seen as a proxy for comorbidities, a barrier for

For peer review only - http://bmjopen.bmj.com/site/about/guidelines.xhtml

adherence [35]. Surprisingly in our sample, the presence of arthritis or pain while walking was not an adherence barrier, as described in other studies [36]. However, reported pain levels were low, and bias from under-enrolment of those with severe arthritis is possible.

While exercise is an effective intervention to manage depression [37], depression adversely impacts adherence to exercise interventions, as shown here and in other studies [38]. Measuring participants' depression levels, an established adherence barrier, before commencing an intervention may help predict subsequent adherence and also allow for the tailoring of the intervention and support of participants accordingly.

The third factor which impacted adherence in this study was risk of accelerated functional decline. Those at high risk were limited by the burden of already deteriorating health, as shown previously [10], while those at moderate risk may have understood the urgency to undertake preventative measures and were still capable of doing so [39]. Previous exercise programmes in participants with cardiovascular conditions have shown that education and awareness regarding one's health can have a positive effect on intervention adherence levels [40]. Participants within our trial were informed of their risk profile and had undergone detailed assessment, which highlighted their balance and strength deficits, and could have acted as a 'prompt' to adhere to the intervention.

When enrolling participants in an intervention programme, assessing physical and psychological adherence barriers can help tailor the intervention delivery to suit participants' preferences and needs [38]. This provides an opportunity to identify non-adherers early and target known barriers, for example, by applying behaviour change techniques to address psychological barriers, such as poor self-efficacy or poor motivation (or depression) [32], or by multidisciplinary interventions to manage pain and comorbidities better.

For peer review only - http://bmjopen.bmj.com/site/about/guidelines.xhtml

Estimated association of adherence

Adherence to an intervention programme influences the intervention dose, which for physical activity interventions can lead to an improvement in physical outcome measures [17, 41]. Predictive modelling showed that higher adherence levels were significantly associated with improved subjective health and function (LLFDI), as well as with improved behavioural complexity scores, gait speed and depressive symptoms. These results are in line with the previous reporting of balance training improving self-reported function in community-dwelling older adults [42], as well as exercise improving depression levels [43] and increasing gait speed [44]. However, several other physical outcome measures remained unchanged, despite higher estimated adherence levels. These inconclusive findings are similar to those by Hughes et al. [3], who demonstrated that even interventions that focus specifically on improved adherence did not achieve meaningful change in clinical outcome measures. Future trials need to understand who within their patient population will adhere, and why, and which adherence barriers can be addressed and modified. Further work is also needed to better understand whether adhering to the dose, type or exercise intensity prescribed has the same or different impact on outcome.

The analysis presented here spans the first six months of the intervention period. Overall, the adherence rates were low, with an average EARS score of 16.75 points at six months in the eLiFE group and 15.29 in the aLiFE group. These results are in line with findings from other technology-supported trials, which have shown adherence rates to drop as time passes, with only 25% of participants still adhering at the end of a 48-week home-exercise programme [45]. The slightly higher adherence rates in eLiFE could be due to the functionalities available within the app, which included daily reminders to complete the exercise, motivating messaging and alternative exercise suggestions.

For peer review only - http://bmjopen.bmj.com/site/about/guidelines.xhtml

Within the intervention period, participants in eLiFE and aLiFE received four or six home visits respectively. Participants were encouraged to integrate their activities into everyday routines, without supervision. Although unsupervised activities have been shown to improve self-reported adherence [46], this did not translate into an improvement in clinical outcome measures. A possible explanation is that while better adherence is reported, the quality, accuracy, and intensity of the activity is not ideal when unsupervised, limiting its benefits. Further work incorporating technological measures of adherence are needed to document accurately the quality and fidelity of the activity undertaken in an unsupervised setting. Beyond documenting exercise execution, an ICT platform could provide feedback to participants about how and what to improve during the intervention programme. Simultaneously, health care providers and researchers could access this information and use telecommunication to increase adherence, optimise exercise accuracy/quality, and increase the likelihood of achieving an improvement in clinical outcomes.

Strength and limitations

A strength of this study was that the activity programme offered to the participants was not detached from daily life, but instead integrated into existing routines. Providing an opportunity to be more active has been labelled a key element to achieving better adherence rates both with [47] and without [7] the use of technology. Within this trial, the intervention was personalised and adapted to the individual's capabilities, similar to approaches by other researchers who individualised interventions integrated into existing routines [48, 49]. Within our study [15], different behaviour change techniques were applied and combined to improve adherence , as this has been identified as an essential intervention component to promote sustainable changes in activity habits [33]. Future studies should evaluate technology-delivered methods which apply different behaviour change techniques to unravel the added benefit of both components.

For peer review only - http://bmjopen.bmj.com/site/about/guidelines.xhtml

During analysis, adherence was treated as a continuous variable, which fulfils the fifth assumption (exclusion restriction) in so called "complier average causal effect analysis" [26-28]. Using the continuous EARS score to quantify adherence can capture the effect of proportionate adherence.

Several limitations of this study are acknowledged. The estimate of the association of adherence only allows us to determine participation levels are associated with better outcomes and it does not estimate the causal effects of the treatment since there is no comparison with a comparable group. As the intention-to-treat analysis showed no difference in effect between groups, a suitable adherer "average causal effect analysis" was not possible. Two different treatment modes are compared based on adherence levels. It was not possible to assess whether adherence to eLiFE has greater implications on clinical outcomes than adherence to aLiFE. However, both intervention modes apply the same activity framework, and analysis to determine the estimated effect of adherence was defined *a priori*. Future work should consider further subgroup analysis to understand the adherence to different intervention methods better. Lastly, monthly measurement of adherence could have acted as a prompt to complete the planned activities. Within our study all participants were asked to report adherence regardless of group allocation, therefore if adherence was influenced by monthly questioning this was consistent for all participants.

Implications for future research and clinical practice

Group allocation did not impact adherence in this study; however, research [50] has shown that adherence can be increased when patients can select the intervention mode. Offering patients a choice of intervention (patient preference) it could impact adherence rates without compromising evidence-based interventions [51].

For peer review only - http://bmjopen.bmj.com/site/about/guidelines.xhtml

To encourage higher rates of adherence, barriers such as depressive symptoms or polypharmacy, could be addressed as part of a pre-intervention to enhance the ability to test new interventions.

In future trials, we should consider measuring baseline characteristics that affect adherence levels. This may include variables not generally considered for standard data collection, such as social support to support interventions, what exercise programmes have previously been explored, and outcome expectations [15]. With improved baseline prediction of adherence and patient preference interventions, subsequent interventions can be more accurately assessed.

Conclusion: Physical activity adherence was associated with better lower extremity function and physical behavioural complexity. Barriers to adherence should be addressed pre-intervention, to enhance intervention efficacy. Further research is needed to unravel the impact of behaviour change techniques embedded into technology-delivered activity interventions on adherence.

Funding: This work was supported by the EU funded project PreventIT (2016–2018, grant number 689238) responding to the Horizon 2020, Personalized Health and Care call PHC-21: Advancing active and healthy aging with ICT: Early risk detection and intervention. The EU was not actively responsible or involved in the study design, collection, management, analysis, or interpretation of data. The writing of reports and the decision to submit for publication is not authorized by the EU.

For peer review only - http://bmjopen.bmj.com/site/about/guidelines.xhtml

Acknowledgement: We thank all the participants in the trial, additional medical staff, assessors and instructors at the three clinical sites, and all partners in the consortium for their support, assistance, and input throughout the different phases of the project.

Author Contribution: KT, EB, HHH, ABM, SM, KA, LC, MP, CT, BV, JH and CB designed research; ASM, KT, EB, HHH, KG and AP conducted research; ASM, KT, KG and AP analysed data, performed statistical analysis and interpreted outcomes; ASM, KT and EB prepared the manuscript; ASM, KT, EB, BV, JH and CB had primary responsibility for final content; all authors read and approved the final version of the manuscript.

Data statement

The datasets presented in this article are not readily available because the datasets for this study are not open accessible to all, but only shared between project partners due to ethical approval guidelines in the three clinical sites conducting the multicenter trial, and cannot be shared until 10 years after the end of the project. Requests to access the datasets should be directed to kristin.taraldsen@ntnu.no.

Conflicts of interests

SM and LC own a share in the spin-off company of the University of Bologna, mHealth Technologies srl. AZ was funded by the Doxee s.p.a. during the conduction of the trial, and by CINECA after the end of the project.

The remaining authors declare that the research was conducted in the absence of any commercial or financial relationships that could be construed as a potential conflict of interest.

Patient and Public involvement

For peer review only - http://bmjopen.bmj.com/site/about/guidelines.xhtml

Participants from within the target group were involved in the design of the eLiFE app during the pilot study which formed part of PreventIT.

For peer review only

For peer review only - http://bmjopen.bmj.com/site/about/guidelines.xhtml

References

1. Sherrington, C., et al., *Exercise to prevent falls in older adults: an updated systematic review and meta-analysis.* British journal of sports medicine, 2016. **ePub**(ePub): p. ePub-ePub.
2. Nyman, S.R. and C.R. Victor, *Older people's participation in and engagement with falls prevention interventions in community settings: an augment to the Cochrane systematic review.* Age Ageing, 2012. **41**(1): p. 16-23.
3. Hughes, K.J., et al., *Interventions to improve adherence to exercise therapy for falls prevention in community-dwelling older adults: systematic review and meta-analysis.* Age Ageing, 2019. **48**(2): p. 185-195.
4. Organisation, W.H., *Adherence to long-term therapies*. 2003: Geneva.
5. Aronson, J.K., *Compliance, concordance, adherence.* Br J Clin Pharmacol, 2007. **63**(4): p. 383-4.
6. Hawley-Hague, H., et al., *Review of how we should define (and measure) adherence in studies examining older adults' participation in exercise classes.* BMJ Open, 2016. **6**(6): p. e011560.
7. Clemson, L., et al., *Integration of balance and strength training into daily life activity to reduce rate of falls in older people (the LiFE study): randomised parallel trial.* BMJ, 2012. **345**: p. e4547.
8. Weber, M., et al., *Feasibility and Effectiveness of Intervention Programmes Integrating Functional Exercise into Daily Life of Older Adults: A Systematic Review.* Gerontology, 2018. **64**(2): p. 172-187.
9. Mikolaizak, A.S., et al., *Adherence to a multifactorial fall prevention program following paramedic care: Predictors and impact on falls and health service use. Results from an RCT a priori subgroup analysis.* Australas J Ageing, 2018. **37**(1): p. 54-61.
10. Picorelli, A.M., et al., *Adherence to exercise programs for older people is influenced by program characteristics and personal factors: a systematic review.* J Physiother, 2014. **60**(3): p. 151-6.
11. Knox, C.R., et al., *Treatment compliance and effectiveness of a cognitive behavioural intervention for low back pain: a complier average causal effect approach to the BeST data set.* BMC Musculoskelet Disord, 2014. **15**: p. 17.
12. Taraldsen, K., et al., *Protocol for the PreventIT feasibility randomised controlled trial of a lifestyle-integrated exercise intervention in young older adults.* BMJ Open, 2019. **9**(3): p. e023526.
13. Taraldsen, K., et al., *Digital Technology to Deliver a Lifestyle-Integrated Exercise Intervention in Young Seniors—The PreventIT Feasibility Randomized Controlled Trial.* 2020. **2**(10).
14. Schwenk, M., et al., *The Adapted Lifestyle-Integrated Functional Exercise Program for Preventing Functional Decline in Young Seniors: Development and Initial Evaluation.* Gerontology, 2019. **65**(4): p. 362-374.
15. Boulton, E., et al., *Implementing behaviour change theory and techniques to increase physical activity and prevent functional decline among adults aged 61-70: The PreventIT project.* Prog Cardiovasc Dis, 2019. **62**(2): p. 147-156.
16. Newman-Beinart, N.A., et al., *The development and initial psychometric evaluation of a measure assessing adherence to prescribed exercise: the Exercise Adherence Rating Scale (EARS).* Physiotherapy, 2017. **103**(2): p. 180-185.
17. Fairhall, N., et al., *A multifactorial intervention for frail older people is more than twice as effective among those who are compliant: complier average causal effect analysis of a randomised trial.* J Physiother, 2017. **63**(1): p. 40-44.
18. Jette, A.M., et al., *Late life function and disability instrument: I. Development and evaluation of the disability component.* J Gerontol A Biol Sci Med Sci, 2002. **57**(4): p. M209-16.
19. Paraschiv-Ionescu, A., et al., *Concern about Falling and Complexity of Free-Living Physical Activity Patterns in Well-Functioning Older Adults.* Gerontology, 2018. **64**(6): p. 603-611.
20. Haley, S.M., et al., *Late Life Function and Disability Instrument: II. Development and evaluation of the function component.* J Gerontol A Biol Sci Med Sci, 2002. **57**(4): p. M217-22.

For peer review only - http://bmjopen.bmj.com/site/about/guidelines.xhtml

21. Paraschiv-Ionescu, A., et al., *Barcoding human physical activity to assess chronic pain conditions.* PLoS One, 2012. **7**(2): p. e32239.
22. Paraschiv-Ionescu, A., et al., *Quantifying dimensions of physical behavior in chronic pain conditions.* J Neuroeng Rehabil, 2016. **13**(1): p. 85.
23. Zhang, W., et al., *Complexity of Daily Physical Activity Is More Sensitive Than Conventional Metrics to Assess Functional Change in Younger Older Adults.* Sensors (Basel), 2018. **18**(7).
24. Jonkman, N.H., et al., *Predicting Trajectories of Functional Decline in 60- to 70-Year-Old People.* Gerontology, 2018. **64**(3): p. 212-221.
25. Allison, P.D., *Missing Data*. 2001, Thousand Oaks, CA: Sage University Press.
26. Sun, X., et al., *Is a subgroup effect believable? Updating criteria to evaluate the credibility of subgroup analyses.* BMJ, 2010. **340**: p. c117.
27. Stuart, E.A., et al., *Estimating intervention effects of prevention programs: accounting for noncompliance.* Prev Sci, 2008. **9**(4): p. 288-98.
28. Herbert, R.D., J. Kasza, and K. Bo, *Analysis of randomised trials with long-term follow-up.* BMC Med Res Methodol, 2018. **18**(1): p. 48.
29. Aiken, L.S. and S.G. West, *Multiple Regression: Testing and Interpreting Interactions*. 1991, Newbury Park: Sage Publications.
30. Peugh, J.L., et al., *Beyond intent to treat (ITT): A complier average causal effect (CACE) estimation primer.* J Sch Psychol, 2017. **60**: p. 7-24.
31. Lamb, S.E., et al., *Development of a common outcome data set for fall injury prevention trials: the Prevention of Falls Network Europe consensus.* J Am Geriatr Soc, 2005. **53**(9): p. 1618-22.
32. French, D.P., et al., *Which behaviour change techniques are most effective at increasing older adults' self-efficacy and physical activity behaviour? A systematic review.* Ann Behav Med, 2014. **48**(2): p. 225-34.
33. Michie, S., et al., *Effective techniques in healthy eating and physical activity interventions: a meta-regression.* Health Psychol, 2009. **28**(6): p. 690-701.
34. Shier, V., E. Trieu, and D.A. Ganz, *Implementing exercise programs to prevent falls: systematic descriptive review.* Inj Epidemiol, 2016. **3**(1): p. 16.
35. Saadat, Z., et al., *Adherence to Antihypertensives in Patients With Comorbid Condition.* Nephrourol Mon, 2015. **7**(4): p. e29863.
36. Marks, R. and J.P. Allegrante, *Chronic osteoarthritis and adherence to exercise: a review of the literature.* J Aging Phys Act, 2005. **13**(4): p. 434-60.
37. Stanton, R. and P. Reaburn, *Exercise and the treatment of depression: a review of the exercise program variables.* J Sci Med Sport, 2014. **17**(2): p. 177-82.
38. Morey, M., et al., *From Supervised to Unsupervised Exercise: Facors associated with Exercise Adherence.* Journal of Aging and Physical Activity, 2003. **11**: p. 351-368.
39. Iliou, M.C., et al., *Cardiac rehabilitation in patients with pacemakers and implantable cardioverter defibrillators.* Monaldi Arch Chest Dis, 2016. **86**(1-2): p. 756.
40. Alkerwi, A., et al., *Adherence to physical activity recommendations and its associated factors: an interregional population-based study.* J Public Health Res, 2015. **4**(1): p. 406.
41. van Gool, C.H., et al., *Effects of exercise adherence on physical function among overweight older adults with knee osteoarthritis.* Arthritis Rheum, 2005. **53**(1): p. 24-32.
42. Roaldsen, K.S., et al., *Task-specific balance training improves self-assessed function in community-dwelling older adults with balance deficits and fear of falling: a randomized controlled trial.* Clin Rehabil, 2014. **28**(12): p. 1189-97.
43. Aguinaga, S., et al., *Home-Based Physical Activity Program Improves Depression and Anxiety in Older Adults.* J Phys Act Health, 2018. **15**(9): p. 692-696.
44. Brach, J.S. and J.M. Vanswearingen, *Interventions to Improve Walking in Older Adults.* Curr Transl Geriatr Exp Gerontol Rep, 2013. **2**(4).
45. Gschwind, Y.J., et al., *The effect of sensor-based exercise at home on functional performance associated with fall risk in older people - a comparison of two exergame interventions.* Eur Rev Aging Phys Act, 2015. **12**: p. 11.

For peer review only - http://bmjopen.bmj.com/site/about/guidelines.xhtml

46. Lacroix, A., et al., *Effects of Supervised vs. Unsupervised Training Programs on Balance and Muscle Strength in Older Adults: A Systematic Review and Meta-Analysis.* Sports Med, 2017. **47**(11): p. 2341-2361.
47. Wu, G., et al., *Comparison of telecommunication, community, and home-based Tai Chi exercise programs on compliance and effectiveness in elders at risk for falls.* Arch Phys Med Rehabil, 2010. **91**(6): p. 849-56.
48. Lord, S.R., et al., *The effect of an individualized fall prevention program on fall risk and falls in older people: a randomized, controlled trial.* J Am Geriatr Soc, 2005. **53**(8): p. 1296-304.
49. Boongird, C., et al., *Effects of a simple home-based exercise program on fall prevention in older adults: A 12-month primary care setting, randomized controlled trial.* Geriatr Gerontol Int, 2017. **17**(11): p. 2157-2163.
50. van Overbeeke, E., et al., *Factors and situations influencing the value of patient preference studies along the medical product lifecycle: a literature review.* Drug Discov Today, 2019. **24**(1): p. 57-68.
51. Yardley, L., et al., *Attitudes and beliefs that predict older people's intention to undertake strength and balance training.* The journals of gerontology. Series B, Psychological sciences and social sciences, 2007. **62**(2): p. P119-25.

For peer review only

For peer review only - http://bmjopen.bmj.com/site/about/guidelines.xhtml

Table 1: Demographics

	aLiFE n = 59	eLiFE n = 61
**Age, years (mean, SD)**	66.19 (2.32)	66.43 (2.33)
**Gender, female (n, %)**	30 (50.8%)	33 (54.1%)
**Living alone (n, %)**	21 (36.7%)	18 (29.5%)
**Pain during rest, 0-10, (median, IQR)**	1.0 (1.0-3.0)	1.0 (1.0-3.0)
**Pain during walking, 0-10, (mean, SD)**	2.5 (1.0-4.0)	2.0 (1.0-4.5)
**Falls in past year (n, %)**		
**0**	53 (91.4%)	51 (83.6%)
**1**	4 (6.9%)	10 (16.4%)
**2+**	1 (1.7%)	0 (0)
**Economic satisfaction (n, %)**		
- **Good**	23 (39.7%)	23 (37.7%)
- **Sufficient**	22 (37.9%)	31 (50.8%)
- **Poor/bad**	13 (22.4%)	7 (11.5%)
**Total number comorbidities, (median, IQR)**	2.0 (1.0-4.0)	2.0 (1.0-3.0)
**Total number medication, (median, IQR)**	2.0 (1.0-4.0)	2.0 (1.0- 3.0)
**Confirmed arthritis (n,%)**	19 (32.8%)	18 (29.5%)
**Confirmed cardio-vascular disease (n,%)**	9 (15.5%)	14 (23.0%)
**CES-D score (median, IQR), 0-60**	8.0 (3.0-14.0)	6.5 (3.25-11.0)
**Moderate risk of functional decline (n,%)**	7 (11.9%)	6 (10%)
**LLFDI score (mean, SD)0-100**		
**Functional (overall)**	73.7 (12.9)	73.1(10.6)

For peer review only - http://bmjopen.bmj.com/site/about/guidelines.xhtml

**Lower extremities basic**	85.3 (15.3)	83.6 (12.7)
**Lower extremities advanced**	70.5 (15.7)	71.5 (15.4)
**Upper extremities**	88.0 (12.7)	87.7 (12.1)
**Disability frequency**	52.2 (4.6)	50.5 (4.0)
**Disability limitation (median, IQR) 0-100**	84 (72.6 to 100)	80 (72 to 100)
**Behavioural complexity score (mean, SD)**	0.347 (0.123)	0.374 (0.119)

IQR, interquartile range; SD, standard deviation; CES-D, Center for Epidemiological Studies Depression scale; LLFDI, Late-Life Function and Disability Index.

For peer review only

For peer review only - http://bmjopen.bmj.com/site/about/guidelines.xhtml

Table 2: Reported adherence levels

		Monthly adherence reporting				EARS score at six-months follow-up (/24) (mean, SD) (n=106)
**Adherence level**		Full adherence	Partial adherence	Non-adherence	Missing	
Treatment mode						
	**All**	26	59	21	14	16.02 (5.12)
	**eLiFE**	15	27	10	8	16.75 (5.01)
	**aLiFE**	11	32	11	*6*	15.29 (5.18)
Mean (min,max) EARS score	All	20.3 (12,24)	15.6 (7,24)	14.1 (4,22)		
	eLIFE	20.6 (12,24)	16.0 (7,24)	16.0 (6,14)		
	aLIFE	19.9 (16,24)	15.3 (8,24)	12.2 (4,22)		

Missing: 3 participants failed to report their monthly adherence repeatedly, beyond where imputation was possible, further 11 withdrew during follow-up.

EARS, Exercise adherence rating scale; SD, standard deviation.

For peer review only

Table 3: Determinants of adherence level (measured using the Exercise Adherence Rating Scale).
Multivariate linear regression (n= 120, aLiFE and eLiFE participants)

	**Univariate**			**Multivariate ***		
	B (SE)	ß	p-value	B (SE)	ß	p-value
**Sociodemographic**						
**Gender: female**	1.56 (1.01)	0.14	.153			
**Age, (years) §**	-0.12 (0.22)	-0.05	.587			
**Satisfaction with economic situation §**	-0.01 (0.72)	-0.002	.987			
**Living status (alone)~**	2.12 (1.06)	0.20	**.048**			
**Years of education ~**	0.003 (0.11)	0.003	.978			
**Medical history**						
**Number of medication§**	-0.61 (0.21)	-0.29	**.003**	**-0.45 (0.21)**	**-0.21**	**.033**
**Been diagnosed/treated for arthritis ǂ**	-2.10 (1.08)	-0.19	.056			
**Been diagnosed/treated for cardiovascular diseases ǂ**	-0.47 (1.30)	-0.04	.721			
**Cognition, affect and well-being**						
**MOCA score ~**	0.27 (0.27)	0.10	.337			
**CES-D score ~**	-0.20 (0.08)	-0.25	**.011**	**-0.20 (0.07)**	**-0.25**	**.009**
**Short Form 12 (SF-12) ~**	0.48 (0.17)	0.28	**.006**			
**Physical ability and mobility ǂ**						
**Gait speed – usual pace (4m walk)**	5.56 (2.29)	0.24	**.017**			
**Gait speed – usual pace (7m walk)**	4.30 (2.02)	0.21	**.036**			
**Fast gait speed – fast pace (7m walk)**	1.82 (1.02)	0.18	.077			
**CBMS (/96)**	0.11 (0.04)	0.28	**.004**			
**8 Level Balance test (/8)ǂ**	0.93 (0.52)	0.18	.077			
**Cadence, mean (SD)**	0.07 (0.11)	0.07	.517			
**Complexity, mean (SD)**	-0.10 (4.13)	-0.002	.981			
**Subjective health rating**						
**Pain while walking, VAS~**	-0.31 (0.22)	-0.14	.151			
**Pain during rest, VAS ~**	-0.03 (0.26)	-0.01	.915			
**LLFDI disability frequency (/100)**	0.17 (0.12)	0.14	.150			
**LLFDI disability limitation (/100)**	0.08 (0.04)	0.21	**.032**			

For peer review only - http://bmjopen.bmj.com/site/about/guidelines.xhtml

**LLFDI function total (/100)**	0.10 (0.04)	0.24	**.002**			
**LLFDI function UE**	0.08 (0.03)	0.19	**.015**			
**LLFDI function BLE**	0.09 (0.04)	0.25	**.011**			
**LLFDI function ALE**	0.10 (0.03)	0.28	**.004**			
**<u>Other potential adherence mediators</u>**						
**Group allocation aLiFE vs eLiFE**	-1.45 (1.01)	-0.14	.153			
**Risk screening moderate category**	-0.08 (0.02)	-0.03	**.006**	**-0.06 (0.03)**	**-0.21**	**.035**

~ Factors which influence adherence; * only variables retained in the final model presented; ǂ Strength and balance deficits known to influence adherence; § Know risk factors for functional decline; SD, standard deviation; MOCA, Montreal Cognitive Assessment; CES-D, Center for Epidemiological Studies Depression scale; CBMS, community balance and mobility scale; VAS, visual analogue scale; LLFDI, Late-Life Function and Disability Index; UE, upper extremity; BLE, basic lower extremity; ALE, advanced lower extremity; CG, control group;

For peer review only

For peer review only - http://bmjopen.bmj.com/site/about/guidelines.xhtml

Table 4: **Estimated association of adherence and group allocation at follow-up** (aLiFE and eLiFE; n=120)

	**Unstandardized B**	**Coefficients Std. Error**	**Stnβ**	**95% CI**	**p Value**
**Primary outcome measures**					
**Late Life Function and Disability Index (LLFDI)**					
**LLFDI disability frequency**					
**Baseline score**	.677	.065	.716	.549 to .80	.000
**Group allocation**	.872	.553	.105	-.226 to 1.970	.118
**Predicted adherence level**	.080	.127	.042	-.173 to .333	.533
**LLFDI disability limitation (/100)**					
**Baseline score**	.663	.081	.621	.502 to .824	.000
**Group allocation**	-.487	1.968	-.017	-4.390 to 3.417	.805
**Predicted adherence level**	.663	.081	.621	.346 to 2.393	**.009**
**LLFDI function total components**					
**Baseline score**	.779	.064	.760	.652 to .906	.000
**Group allocation**	-1.354	1.260	-.057	-3.854 to 1.146	.285
**Predicted adherence level**	.797	.344	.144	.114 to 1.480	**.023**
**LLFDI UE**					
**Baseline score**	.624	.074	.623	.478 to .771	.000
**Group allocation**	-.667	1.694	-.027	-4.028 to 2.693	.694
**Predicted adherence level**	1.113	.418	.197	.285 to 1.942	**.009**
**LLFDI BLE**					
**Baseline score**	.632	.076	.631	.481 to .784	.000
**Group allocation**	-1.994	1.788	-.072	-5.540 to 1.552	.267
**Predicted adherence level**	1.304	.486	.204	.339 to 2.268	**.009**
**LLFDI ALE**					
**Baseline score**	.868	.066	.780	.736 to .999	.000
**Group allocation**	-1.934	1.763	-.057	-5.431 to 1.562	.275
**Predicted adherence level**	1.000	.467	.127	.074 to 1.926	**.035**
**Behavioural complexity**					
**Complexity, mean**					
**Baseline score**	.178	.076	.235	.028 to .329	.021
**Group allocation**	-.012	.019	-.063	-.049 to .025	.525
**Predicted adherence level**	.008	.004	.185	-.001 to .017	.068
**Complexity, median (IQR)**					
**Baseline score**	.209	.076	.270	.057 to .360	.008

For peer review only

For peer review only - http://bmjopen.bmj.com/site/about/guidelines.xhtml

**Group allocation**	-.007	.019	-.035	-.045 to .031	.726
**Predicted adherence level**	.008	.004	.186	-.045 to .017	.063
**Secondary outcome measures**					
**Cognition, affect and well-being**					
**MOCA score**					
**Baseline score**	.590	.090	.558	.411 to .769	.000
**Group allocation**	.190	.328	.049	-.461 to .842	.563
**Predicted adherence level**	-.023	.077	-.026	-.176 to .129	.762
**CES-D score**					
**Baseline score**	.426	.087	.444	.253 to .598	.000
**Group allocation**	-.586	.903	-.048	-2.377 to 1.205	.518
**Predicted adherence level**	-.889	.257	-.313	-1.399 to -.380	**.001**
**Short Form 12 (SF-12)**					
**Baseline score**	.500	.102	.467	.297 to .703	.000
**Group allocation**	-.990	.517	-.158	-2.017 to .037	.059
**Predicated adherence level**	.231	.138	.160	-.042 to .505	.097
**Physical ability and mobility** ǂ					
**Usual gait speed (7m walk)**					
**Baseline score**	.621	.057	.717	.507 to .735	.000
**Group allocation**	.041	.027	.097	-.012 to .095	.128
**Predicted adherence level**	.014	.007	.141	.001 to .027	**.036**
**CBMS (/96)**					
**Baseline score**	.847	.073	.796	.702 to .992	.000
**Group allocation**	1.039	1.599	.038	-2.134 to 4.212	.517
**Predicated adherence level**	.206	.431	.033	-.648 to 1.061	.633
**8 Level Balance test**					
**Baseline score**	.467	.116	.396	.237 to .697	.000
**Group allocation**	.254	.213	.111	-.168 to .677	.235
**Predicted adherence level**	.047	.051	.089	-.053 to .147	.356

MOCA, Montreal Cognitive Assessment; CES-D, Center for Epidemiological Studies Depression scale; CBMS, community balance and mobility scale; VAS, visual analogue scale; LLFDI, Late-Life Function and Disability Index; UE, upper extremity; BLE, basic lower extremity; ALE, advanced lower extremity;

For peer review only - http://bmjopen.bmj.com/site/about/guidelines.xhtml

Reporting checklist for randomised trial.

Based on the CONSORT guidelines.

Instructions to authors

Complete this checklist by entering the page numbers from your manuscript where readers will find each of the items listed below.

Your article may not currently address all the items on the checklist. Please modify your text to include the missing information. If you are certain that an item does not apply, please write "n/a" and provide a short explanation.

Upload your completed checklist as an extra file when you submit to a journal.

In your methods section, say that you used the CONSORTreporting guidelines, and cite them as:

Schulz KF, Altman DG, Moher D, for the CONSORT Group. CONSORT 2010 Statement: updated guidelines for reporting parallel group randomised trials

For peer review only

		Reporting Item	Page Number
**Title and Abstract**			
Title	#1a	Identification as a randomized trial in the title.	1
Abstract	#1b	Structured summary of trial design, methods, results, and conclusions	2
**Introduction**			

Background and objectives	#2a	Scientific background and explanation of rationale	4
Background and objectives	#2b	Specific objectives or hypothesis	5
**Methods**			
Trial design	#3a	Description of trial design (such as parallel, factorial) including allocation ratio.	5
Trial design	#3b	Important changes to methods after trial commencement (such as eligibility criteria), with reasons	n/a
Participants	#4a	Eligibility criteria for participants	6
Participants	#4b	Settings and locations where the data were collected	6
Interventions	#5	The experimental and control interventions for each group with sufficient details to allow replication, including how and when they were actually administered	6
Outcomes	#6a	Completely defined prespecified primary and secondary outcome measures, including how and when they were assessed	7
Sample size	#7a	How sample size was determined.	6
Sample size	#7b	When applicable, explanation of any interim analyses and stopping guidelines	n/a

For peer review only

Randomization - Sequence generation 6	#8a	Method used to generate the random allocation sequence.	
Randomization - Sequence generation 6	#8b	Type of randomization; details of any restriction (such as blocking and block size)	
Randomization - Allocation concealment mechanism	#9	Mechanism used to implement the random allocation sequence (such as sequentially numbered containers), describing any steps taken to conceal the sequence until interventions were assigned	6
Randomization - Implementation	#10	Who generated the allocation sequence, who enrolled participants, and who assigned participants to interventions	6
Blinding	#11a	If done, who was blinded after assignment to interventions (for example, participants, care providers, those assessing outcomes) and how.	6
Blinding	#11b	If relevant, description of the similarity of interventions	n/a
Statistical methods	#12a	Statistical methods used to compare groups for primary and secondary outcomes	10
Statistical methods	#12b	Methods for additional analyses, such as subgroup analyses and adjusted analyses	10

For peer review only

Outcomes	#6b	Any changes to trial outcomes after the trial commenced, with reasons	n/a
**Results**			
Participant flow diagram (strongly recommended)	#13a	For each group, the numbers of participants who were randomly assigned, received intended treatment, and were analysed for the primary outcome	11
Participant flow	#13b	For each group, losses and exclusions after randomization, together with reason	n/a
Recruitment	#14a	Dates defining the periods of recruitment and follow-up	n/a
Recruitment	#14b	Why the trial ended or was stopped	n/a
Baseline data	#15	A table showing baseline demographic and clinical characteristics for each group	24
Numbers analysed	#16	For each group, number of participants (denominator) included in each analysis and whether the analysis was by original assigned groups	11, 24
Outcomes and estimation	#17a	For each primary and secondary outcome, results for each group, and the estimated effect size and its precision (such as 95% confidence interval)	26
Outcomes and estimation	#17b	For binary outcomes, presentation of both absolute and relative effect sizes is recommended	26

For peer review only

Ancillary analyses	#18	Results of any other analyses performed, including subgroup analyses and adjusted analyses, distinguishing pre-specified from exploratory	26, 28
Harms	#19	All important harms or unintended effects in each group (For specific guidance see CONSORT for harms)	n/a
**Discussion**			
Limitations	#20	Trial limitations, addressing sources of potential bias, imprecision, and, if relevant, multiplicity of analyses	17
Interpretation	#22	Interpretation consistent with results, balancing benefits and harms, and considering other relevant evidence	16
Registration	#23	Registration number and name of trial registry	6
**Other Information**			
Protocol	#24	Where the full trial protocol can be accessed, if available	6
Funding	#25	Sources of funding and other support (such as supply of drugs), role of funders	19

The CONSORT checklist is distributed under the terms of the Creative Commons Attribution License CC-BY. This checklist was completed on 06. June 2021 using https://www.goodreports.org/, a tool made by the EQUATOR Network in collaboration with Penelope.ai

For peer review only
